# Apigenin promotes melanogenesis and melanosome transport through the c-KIT/Raf-1/MAPK/CREB pathway in HEMCs

**DOI:** 10.3389/fphar.2025.1572878

**Published:** 2025-04-28

**Authors:** Jinpeng Lv, Duo Meng, Huansha Zhang, Wenhui Xu, Xiaohong An, Chuanwei Yin, Kun Zou, Rongyin Gao

**Affiliations:** ^1^ Jiangsu Provincial Engineering Research Center for Drug Intelligent Manufacturing and Precision Delivery, School of Pharmacy, Changzhou University, Changzhou, China; ^2^ Yunnan Characteristic Plant Extraction Laboratory, Yunnan Yunke Characteristic Plant Extraction Laboratory Co., Ltd., Kunming, China; ^3^ Yunnan Botanee Bio-Technology Group Co., Ltd., Kunming, China; ^4^ Department of Pharmacy, The First People’s Hospital of Changzhou, The Third Affiliated Hospital of Soochow University, Changzhou, China

**Keywords:** pigmentation disorders, apigenin, HEMCs, melanogenesis, melanosome transport, c-KIT/Raf-1/MAPK pathway

## Abstract

**Introduction:**

Apigenin, a natural flavonoid with well-established antioxidant, anticancer, and anti-inflammatory activities, has recently attracted attention for its pigmentation-promoting effects. However, the underlying molecular mechanisms driving its melanogenic activity remain incompletely understood.

**Method:**

To investigate apigenin‘s effects on melanogenesis, human epidermal melanocytes (HEMCs), zebrafish embryos, and human skin explants were treated with apigenin. Melanin content, dendrite formation, and melanosome maturation were evaluated using spectrophotometry and transmission electron microscopy. Key signaling molecules and proteins involved in melanogenesis and melanosome transport were assessed by Western blotting and immunohistochemistry. The role of the c-KIT receptor was further explored through pharmacological inhibition and genetic knockdown approaches. Functional pigmentation was evaluated by assessing UVB-induced DNA damage markers.

**Results:**

Apigenin (10 μM) significantly increased melanin production by 1.8-fold in HEMCs, enhanced dendritic morphology, and promoted stage III–IV melanosome formation. Mechanistically, apigenin induced melanogenesis independently of the MC1R/cAMP/PKA pathway by directly binding to the c-KIT receptor, activating the Raf-1/ERK/RSK cascade, and upregulating MITF. This led to elevated expression of tyrosinase, TRP-1, TRP-2, and melanosome transport proteins Rab27a and Cdc42. Inhibition or knockdown of c-KIT abrogated these effects. In vivo, apigenin restored pigmentation in PTU-induced depigmented zebrafish and increased melanin content by 1.3-fold in human skin explants. Histological analysis confirmed effective melanin transfer to keratinocytes. Additionally, apigenin-treated skin showed reduced UVB-induced DNA damage, indicating enhanced photoprotection.

**Discussion:**

These findings demonstrate that apigenin stimulates melanogenesis through a novel c-KIT-dependent signaling pathway and promotes functional pigmentation with photoprotective benefits. Given its dietary origin, favorable safety profile, and multifaceted mechanisms, apigenin holds promise as a therapeutic agent for vitiligo and a natural pigmentation enhancer for dermatological use.

## Introduction

Melanin is produced in melanosomes within melanocytes and is essential for the coloration of hair, skin, and eyes (Raposo and Marks, 2007; Slominski et al., 2004). Melanocytes distribute melanin to neighboring keratinocytes through dendrites, forming melanin caps that effectively shield the epidermal layer from ultraviolet radiation-induced DNA damage (Allouche et al., 2021; Ando et al., 2012). Disruptions in melanin production not only lead to pigmentary disorders such as vitiligo-an autoimmune condition characterized by melanocyte loss-but may also compromise cutaneous immune defense and increase susceptibility to environmental insults (Telegina et al., 2023).

The process of melanogenesis is tightly regulated by various signaling pathways, with the Melanocytes inducing transcription factor (MITF) serving as the master regulator. MITF not only controls the expression of melanogenic enzymes, including Tyrosinase, tyrosinase-related protein-1 (TRP-1) and tyrosinase-related protein-2 (TRP-2), but also regulates melanosome transport by modulating proteins such as ras-related protein Rab-27A (Rab27a) and cell division cycle 42 (Cdc42) (Kawasaki et al., 2008; Lv et al., 2020). Ultraviolet B (UVB) irradiation activates melanocytes indirectly via keratinocyte-derived factors such as α-melanocyte-stimulating hormone (α-MSH) and stem cell factor (SCF) (Kwon et al., 2017). Binding of α-MSH to the conventional melanocortin 1 receptor (MC1R) leads to the activation of adenylate cyclase and elevated intracellular cyclic adenosine monophosphate (cAMP) levels, which subsequently activate protein kinase A (PKA). PKA translocates into the nucleus to phosphorylate cAMP response element-binding protein (CREB), thereby inducing MITF transcription and promoting pigmentation (Dores and Baron, 2011; Kim and Hyun, 2022; Lv et al., 2024). Similarly, SCF binding to its receptor cellular-KIT (c-KIT) triggers activation of the rapidly accelerated fibrosarcoma-1 (Raf-1)/mitogen-activated protein kinase kinase (MEK)/signal-regulated kinase (ERK) signaling cascade, leading to phosphorylation of downstream transcription factors such as CREB (via p90 ribosomal S6 kinase (RSK)) and MITF (via ERK and RSK), further enhancing melanogenesis (Imokawa et al., 2000; Niwano et al., 2018; Yun et al., 2020).

Apigenin, a dietary flavonoid abundantly found in fruits, vegetables, and traditional Chinese herbs, has gained attention due to its diverse pharmacological activities, including antioxidant, anti-inflammatory, antimicrobial, and anticancer effects (Jin et al., 2022; Liu-Smith and Meyskens, 2016; Woo et al., 2020). Apigenin has been reported to inhibit the invasion and migration of melanoma cells (Cao et al., 2016; Zhao et al., 2017), and to prevent UV-induced skin cancer (Bridgeman et al., 2016). Notably, apigenin demonstrates low toxicity to normal cells and high selectivity for tumor cells. Ye et al. (2011) reported that apigenin promotes melanogenesis in murine melanoma B16F10 cells (Ye et al., 2011). However, its effect on melanogenesis in human melanocytes and skin tissues remains unclear, and the underlying molecular mechanisms have not been elucidated.

Therefore, the objective of this study was to investigate the melanogenic potential of apigenin in human primary melanocytes, zebrafish, and human skin tissues, and to elucidate the signalling pathways involved. Our results demonstrate that apigenin activates the c-KIT/Raf/mitogen-activated protein kinase (MAPK)/CREB signalling cascade, upregulates MITF expression, and enhances melanin production. These findings reveal a novel role of apigenin in pigmentation regulation and suggest its potential utility as a natural agent for treating hypopigmentation disorders.

## Materials and methods

### Reagents

Apigenin (A106675) and mushroom tyrosinase (T128536) were purchased from Aladdin Biochemical Technology (Shanghai, China). Anti-β-actin (AF0003, 1:1000) and Histone H3 (AF0009, 1:1000) antibodies were obtained from Beyotime Biotechnology (Shanghai, China). Antibodies against MITF (ab140606, 1:1000), Tyrosinase (ab180753, 1:1000), TRP-1 (ab235447, 1:1000), TRP-2 (ab221144, 1:1000), and p-MSK1 (ab32190, 1:2000) were purchased from Abcam (Cambridge, UK). Anti-Myosin Va (sc-365986, 1:200), KIF5b (sc-133184, 1:200), Cdc42 (sc-8401, 1:200), Rab27a (sc-74586, 1:200), p-c-KIT (sc-365504, 1:200), c-KIT (sc-13508, 1:200), p-Raf-1 (sc-271929, 1:200), Raf-1 (sc-7267, 1:200), p-p38 (sc-166182, 1:200), p38 (sc-398546, 1:200), p-ERK (sc-7383, 1:200), ERK (sc-514302, 1:200), p-RSK (sc-377526, 1:200), RSK (sc-74575, 1:200), p-MEK (sc-81503, 1:200), MEK (sc-13159, 1:200), MSK1 (sc-518173, 1:200), p-PKA (sc-293036, 1:200), PKA (sc-390548, 1:200), p-CREB (sc-81486, 1:200), CREB (sc-240, 1:200), CRTC1 (sc-271333, 1:200), c-KIT siRNA (sc-29225), Rab 27a siRNA (sc-41834) and Cdc42 siRNA (sc-29256), siRNA Transfection Reagent (sc-29528), siRNA Transfection Medium (sc-36868) were obtained from Santa Cruz Biotechnology (Dallas, United States). The AC inhibitor 2′,5′-dideoxyadenosine (DDA, HY-135878), MC1R antagonist Nonapeptide-1 (N-1A, HY-P0097A), c-KIT receptor inhibitor ISCK03 (HY-101443), and RSK inhibitor BI-D1870 (HY-10510) were purchased from MedChemExpress (New Jersey, United States). ChamQ SYBR quantitative polymerase chain reaction (qPCR) Master Mix (Q311) and HiScript III RT SuperMix for qPCR (+gDNA wiper) (R323) were obtained from Vazyme Biotechnology (Nanjing, China).

### Cell viability assay

Human epidermal melanocytes (HEMCs), obtained from Procell Life Sciences (Wuhan, China), were cultured in Medium 254 supplemented with HMGS-2 at 37°C and 5% CO_2_. Cell viability was assessed using the MTT assay (Gao et al., 2023). We used the MTT Cell Proliferation and Cytotoxicity Assay Kit (C0009S, Beyotime, Shanghai, China) according to the manufacturer’s instructions. Log-phase cells were harvested and seeded in 96-well plates at a density of 1 × 10^5^ cells/mL (200 mL/well) and allowed to adhere overnight in a humidified incubator at 37°C with 5% CO_2_ until reaching 80%–90% confluence. Following medium aspiration and washing with PBS three times, the cells were treated with 200 μL of fresh culture medium containing different concentrations of apigenin (0, 1, 5, 10, and 20 μM). The vehicle control group received medium containing an equivalent volume of Dimethyl Sulfoxide (DMSO) (final concentration <0.1%). After 48 h incubation, supernatants were replaced with MTT working solution (20 μL MTT + 180 μL medium). The plates were incubated at 37°C for an additional 4 h. The medium was then carefully removed, and 150 μL of DMSO was added to each well to dissolve the formazan crystals. The plate was shaken on an orbital shaker at 180 rpm for 10 min to ensure complete dissolution. Absorbance was measured at 570 nm using a microplate reader (TECAN, Switzerland). Each condition was tested in triplicate, and results were expressed as a percentage of the control group.

### Measurement of melanin content

Cells were seeded in a six-well culture plate at a density of 4 × 10^5^ cells per well and incubated overnight to assess melanin content. Control group: The Cells were culture medium without apigenin. Experimental group: Apigenin was added at concentrations of 1, 5, and 10 μM, and the cells were incubated for an additional 48 h. Subsequently, the cells were collected, and total melanin was extracted from the cell pellet by dissolving it in 100 μL of 1 mol/L NaOH solution containing 10% DMSO, followed by incubation at 80°C for 1 h. The concentration of dissolved melanin was measured at 405 nm using a microplate reader.

### Transmission electron microscopy (TEM)

HEMCs was cultured in the presence of apigenin (10 μM) or medium for 48 h. HEMCs were initially fixed overnight in a solution containing 4% glutaraldehyde. Additional fixation was performed with 2% osmium tetroxide at room temperature for 1 h. Dehydration was achieved using a graded ethanol series, followed by embedding in epoxy resin. The embedded samples were sectioned into thin slices and stained sequentially with uranyl acetate and lead citrate. The processed sections were then examined using a Hitachi H-7800 transmission electron microscope (Gao et al., 2023).

### Tyrosinase activity assay

HEMCs were cultured in six-well plates at a density of 1 × 10^5^ cells per well and incubated for 24 h under optimal conditions. The cells were then treated with apigenin at various concentrations and incubated for an additional 48 h. Following treatment, the cells were washed twice with cold PBS and lysed using a cell lysis solution. The supernatant obtained after centrifugation was collected for measurement of tyrosinase activity. To assess cellular tyrosinase activity, a mixture containing 10 μg of protein, 100 μL of PBS (0.1 M, pH 6.8), and 100 μL of 0.1% Levodopa (L-DOPA) was prepared. After incubation for 1 h at 37°C, absorbance was measured at 475 nm using a microplate reader.

To evaluate the effect of apigenin on mushroom tyrosinase activity, different concentrations of apigenin were incubated with 10 units of mushroom tyrosinase and 0.03% L-tyrosine in 100 μL of PBS (0.1 M, pH 6.5). The samples were maintained at 37°C for 10 min, and their absorbance was measured at 475 nm using a microplate spectrophotometer (Lv et al., 2021).

### Separation of cytosolic and nuclear protein fraction

HEMCs with a density of 2 × 10^5^ cells/mL were seeded in 6-well culture plates. After the cell density reached 80%, the cells were subjected to subsequent experiments. Groups were as follows: (1) Ctrl group; (2) ISCK03 group; (3) Apigenin group; (4) ISCK03 +Apigenin group. For the group (4), cells were cultured with 10 mM ISCK03 stimulation 1 h. After 1 h treatment, 10 μM Apigenin+10 mM ISCK03 were co-applied to incubation for 1 h.

We used Nuclear and Cytoplasmic Protein Extraction Kit (P0027, Beyotime, Shanghai, China). The cells were rinsed with PBS, scraped off, and collected by centrifugation. The pellet was resuspended in Cytoplasmic Protein Extraction Reagent A, vortexed for 5 s, and kept on ice for 15 min. Following the addition of Reagent B, the sample was vortexed again for 5 s, kept on ice for 1 min, and centrifuged at 16,000 × g for 5 min at 4°C. The supernatant containing cytoplasmic proteins was transferred to a pre-cooled tube. The remaining pellet was resuspended in nuclear protein extraction reagent containing PMSF, vortexed for 30 s, and incubated on ice with intermittent vortexing every 2 min for 30 min. After centrifugation at 16,000 × g for 10 min at 4°C, the supernatant with nuclear proteins was transferred to pre-cooled tubes.

### Western blot analysis

HEMCs were seeded into 6-well plates at a density of 2 ×10^5^/well grown for 24 h. Apigenin (10 μM) acts on cells at different times. Then, the total protein of the cells is extracted by Cell lysis buffer for Western and IP (P0013, Beyotime, Shanghai, China). Protein lysates were prepared by homogenizing samples in lysis buffer followed by centrifugation at 10,000 × g for 15 min at 4°C. Protein concentrations were quantified using the BCA assay with bovine serum albumin standards (P0012, Beyotime, Shanghai, China). Total protein samples (20 μg) were separated by SDS-PAGE using 10%, 12%, or 15% gels and then transferred onto NC or PVDF membranes using an electroblotting apparatus (Bio-Rad, United States). The membranes were blocked with a TBST solution containing 5% skim milk powder at room temperature for 1.5 h, followed by overnight incubation with the appropriate primary antibodies at 4°C. After washing the membranes four times with TBST, they were incubated with peroxidase-linked secondary antibodies for 1 h at room temperature. Protein bands were detected using an enhanced chemiluminescence system (Lv et al., 2019). Immunoreactive bands were quantified using ImageJ (NIH) software. The expression levels of target proteins were normalized to β-actin, which was used as the internal loading control. Data were expressed as fold changes relative to the control group.

### Reverse transcription (RT)-qPCR

Total RNA was extracted using TRIzol reagent, and RNA concentration and purity were assessed spectrophotometrically. cDNA was synthesized from 1 μg of total RNA using the HiScript III Reverse Transcriptase kit (Vazyme, China) according to the manufacturer’s instructions. qPCR was conducted using SYBR Green chemistry on an Applied Biosystems ABI PRISM machine with pre-validated primer sets. Each sample was analyzed in triplicate. The Ct values were determined during the exponential phase of amplification. GAPDH was used as the internal reference gene. Relative mRNA expression levels were calculated using the 2^−ΔΔCt^ method (Livak and Schmittgen, 2001). Specifically, ΔCt = Ct (target gene) – Ct (GAPDH), and ΔΔCt = ΔCt (experimental group) – ΔCt (control group). The final relative expression = 2^−ΔΔCt^.

The relative expression level of mRNA in the experimental group compared to the control group was calculated. The primer sequences for MITF were: forward primer: 5′-CTC​ACA​GCG​TGT​ATT​TTT​CCC​A-3′, reverse primer: 5′-ACT​TTC​GGA​TAT​AGT​CCA​CGG​AT-3′. For GAPDH, the primer sequences were: forward primer: 5′-GGA​GCG​AGA​TCC​CTC​CAA​AAT-3′, reverse primer: 5′-GGCTGTTGTCATACTTCTCAT-3′.The primer sequences for Tyrosinase were: forward primer: 5′-TCA​TCC​AAA​GAT​CTG​GGC​TAT​GAC​T-3′, reverse primer: 5′-GTG​ACG​ACA​CAG​CAA​GCT​CAC-3′; The primer sequences for TRP-1 were: forward primer: 5′-AAG​GCT​ACA​ACA​AAA​ATC​ACC​AT-3′; reverse primer: 5′-ATT​GAG​AGG​CAG​GGA​AAC​AC-3′; The primer sequences for TRP-2 were: forward primer: 5′-GCA​GCA​AGA​GAT​ACA​CAG​AAG​AA-3′; reverse primer: 5′-TCC​TTT​ATT​GTC​AGC​GTC​AGA-3′.

### Masson-Fontana melanin staining

To detect the melanin pigment, HEMCs were seeded onto a 6 - well plate with 13 mm glass coverslips placed in each well. Each well was filled with 2.0 mL of culture medium consisting of 10% fetal bovine serum. We used α-MSH as a positive control. HEMCs were incubated with 100 nM α-MSH and 10 μM apigenin for 24 h and then were fixed with Formalin (10% neutral buffered) for 20 min.

The Masson-Fontana staining protocol was applied to process both skin samples and melanocytes (Noguchi et al., 2014). The specimens were stained using the Masson-Fontana ammonia-silver method by incubating them in Fontana ammonia-silver solution overnight at 4°C. After rinsing with distilled water, the cells were treated with Hypo solution for 3 min and then re-stained with neutral red dye for 5 min (Lv et al., 2019). Finally, the stained cells were observed under a Nikon Ti2-U microscope.

### Skin explant treatment

The researches on human skin materials were accepted by the local ethic committee. Briefly, foreskins were cut into pieces. These pieces were carefully placed into William’s E medium supplemented with insulin (10 μg/mL), hydrocortisone (10 ng/mL), and L-glutamine (2 mM). The explants were cultured epidermidis-side up within a humidified incubator at 37°C with 5% CO_2_. After 24 h equilibration, experimental groups received either α-MSH (100 nM) or apigenin (10 μM) via basolateral medium supplementation. Following 120 h culture, skin pigmentation was stained with silver ammonium and analyzed using ImageJ.

### Immunohistochemistry (IHC) for gamma H2AX (phospho S139)

IHC for gamma H2AX (phospho S139) was performed following the method outlined in a previous study (Cao et al., 2024). Slides were blocked with a 5% BSA solution for 1 h at 25°C and then incubated with primary antibodies overnight at 4°C. The slides were washed four times with TBST and incubated with the secondary antibody. Finally, the slides were treated with aminoethyl carbazole for sectioning and examined under a microscope.

### Gene silencing protocol

Seed melanocytes in 6-well plates using antibiotic-free culture medium and incubate until reaching 60%–80% confluence prior to transfection. Transfection Complex Preparation: Solution A: Dilute 8 μL siRNA duplex into 100 μL serum-free siRNA Transfection Medium; Solution B: Suspend 8 μL lipid-based siRNA Transfection Reagent in 100 μL serum-free Transfection Medium through slow vortex-free mixing. Combine Solution A with Solution B using gentle pipetting. Allow the siRNA-lipid complexes to stabilize at ambient temperature for 45 min. Add 0.8 mL serum-free Transfection Medium to the preformed complexes and mix by inverted tube rotation. Aspirate existing culture medium and rinse cells twice with pre-warmed PBS to remove residual serum proteins. Maintain cells under standard culture conditions (37°C, 5% CO_2_) for 6 h. Supplement each well with 1 mL complete growth medium containing 20% FBS. Continue incubation for 18–24 h to ensure cellular recovery prior to downstream analyses.

### Phenotype-based evaluation assays using zebrafish

The experiment followed established procedures (Choi et al., 2007; Van Rooijen et al., 2017). Synchronized zebrafish embryos were collected and placed into six-well plates, with 30 embryos per well in 2 mL of medium. Transparent zebrafish were induced from 6 to 35 h post-fertilization with 0.2 mM Propylthiouracil (PTU), serving as the standard positive control (Elsalini and Rohr, 2003). The embryo medium was supplemented with apigenin and incubated for an additional 35 to 60 h. Zebrafish pigmentation was assessed visually using an Olympus SZ61 stereomicroscope. To quantify melanin levels, 30 zebrafish embryos were homogenized and centrifuged at 13,000 rpm for 25 min at 4°C. The melanin was dissolved by heating the pellet in 200 μL of 1 mol/L NaOH solution containing 10% DMSO at 90°C for 1 h. Absorbance at 405 nm was measured using a Tecan Spark microplate reader.

### Immunocytochemistry

The HEMCs were immunostained with anti-MITF after 100 nM α-MSH and 10 μM apigenin treatment 24 h. Regarding the sample of the skin tissue, about 48 h after the 100 nM α-MSH and 10 μM apigenin treatment, the sample were immunostained with anti-Tyrosinase according to the standard immunofluorescence protocol. The nuclei were stained with DAPI (C1005, Beyotime, Shanghai, China). Images were obtained using Nikon Ti2-U microscope.

### Statistical analysis

The data are presented as the mean ± standard error of the mean (SEM). Before performing any statistical tests, the normality of the data was assessed using the Shapiro-Wilk test. If the data were normally distributed, one-way analysis of variance (ANOVA) was used, followed by Tukey’s *post hoc* multiple comparison test. If the data did not follow a normal distribution, a Kruskal–Wallis test was applied, followed by Dunn’s *post hoc* test. Pearson’s correlation coefficient was used to evaluate the relationship between two variables, and the significance of the regression slope was assessed using the t-statistic. All statistical analyses were conducted using GraphPad Prism 9.0 software, with statistical significance set at p < 0.05.

## Results

### Apigenin induced melanogenesis in HEMCs

**FIGURE 1:**
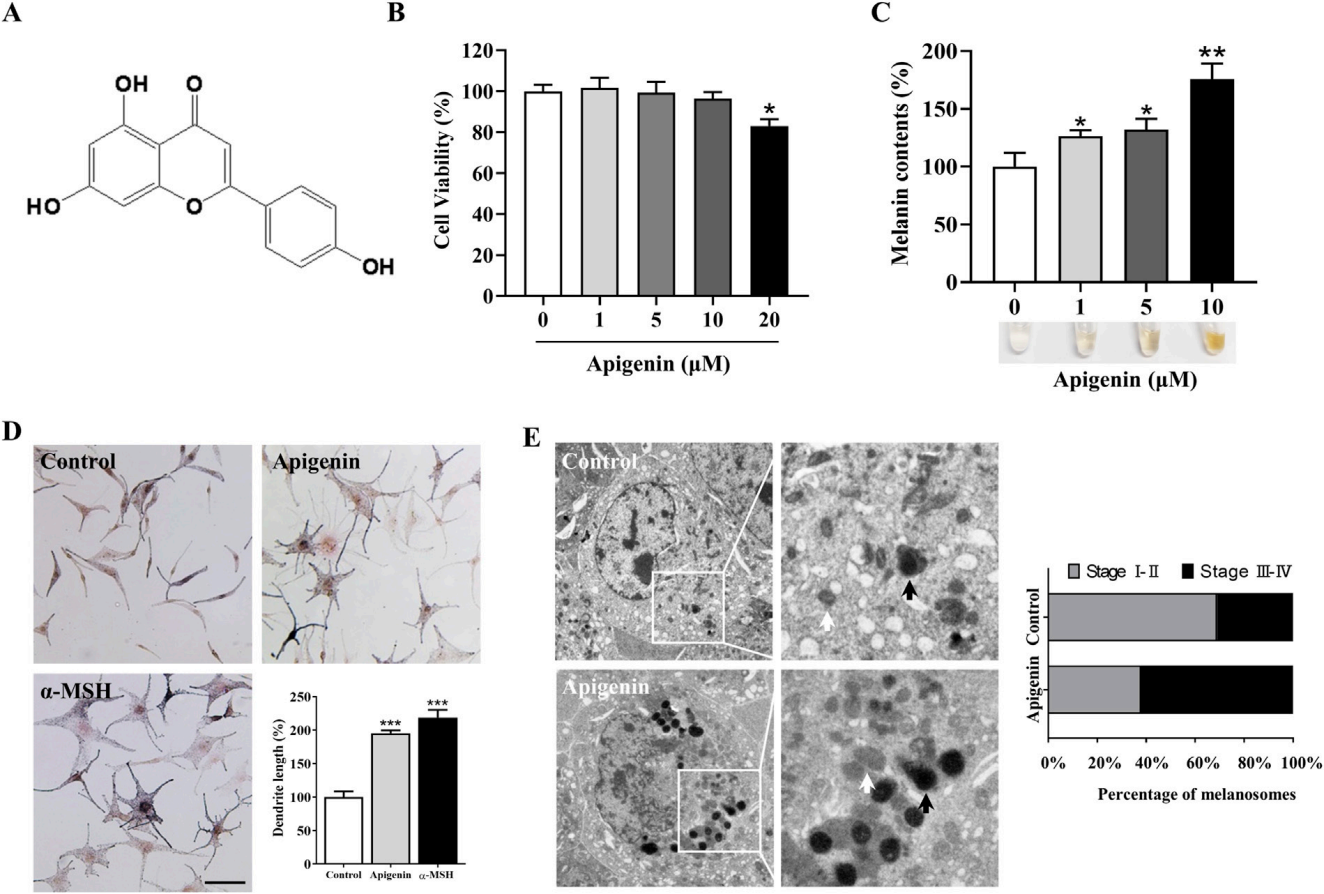
Apigenin induces hyperpigmentation in melanocytes. **(A)** Chemical structure of apigenin. **(B)** HEMCs were treated with various concentrations (0–20 μM) of apigenin for 48 h, and cell viability was assessed using the MTT assay. **(C)** HEMCs were treated with apigenin (0, 1, 5, or 10 μM) for 48 h, and melanin content was measured. **(D)** HEMCs were treated with apigenin (10 μM) or α-MSH (100 nM, positive control) for 48 h, followed by Masson-Fontana ammoniacal silver staining. Scale bar = 10 μm. The total dendrite length per cell was measured, and twenty cells per condition were analyzed in three independent experiments. **(E)** HEMCs were observed using TEM after 48 h of treatment with apigenin (10 μM). White and black arrows indicate stages I–II and III–IV melanosomes, respectively. The percentages of stages I–II and III–IV melanosomes relative to the total number of melanosomes are shown. Data are expressed as the mean ± SEM (n = 3). *p < 0.05, **p < 0.01, and ***p < 0.001 vs. untreated cells. HEMCs, human epidermal melanocytes; α-MSH, α-melanocyte-stimulating hormone; TEM, Transmission electron microscopy.

Apigenin, a flavonoid also known as 4,5,7-trihydroxyflavone, is commonly found in various fruits and vegetables (Li et al., 2018) (Figure 1A). Before assessing the impact of apigenin on melanin production, we first conducted a cell viability assay to evaluate its effects on HEMC growth. As shown in Figure 1B, apigenin (1–10 μM) exhibited no toxicity after 48 h of treatment. Based on these results, we selected concentrations of 1, 5, and 10 μM to investigate apigenin’s effect on melanin production. Figure 1C demonstrates that apigenin significantly enhanced melanin production in HEMCs. Interestingly, Fontana-Masson staining revealed that apigenin not only increased melanin levels but also augmented dendrite formation (Figure 1D). TEM analysis of melanosome development in HEMCs (Figure 1E) showed that apigenin notably increased the prevalence of stage III-IV melanosomes, suggesting it accelerates melanosomal maturation. Collectively, our data indicate that apigenin enhances melanin production and promotes melanosome maturation in HEMCs.

### Apigenin increased cellular tyrosinase activity and the tyrosinase, TRP-1, TRP-2 and MITF expression levels in HEMCs

**FIGURE 2:**
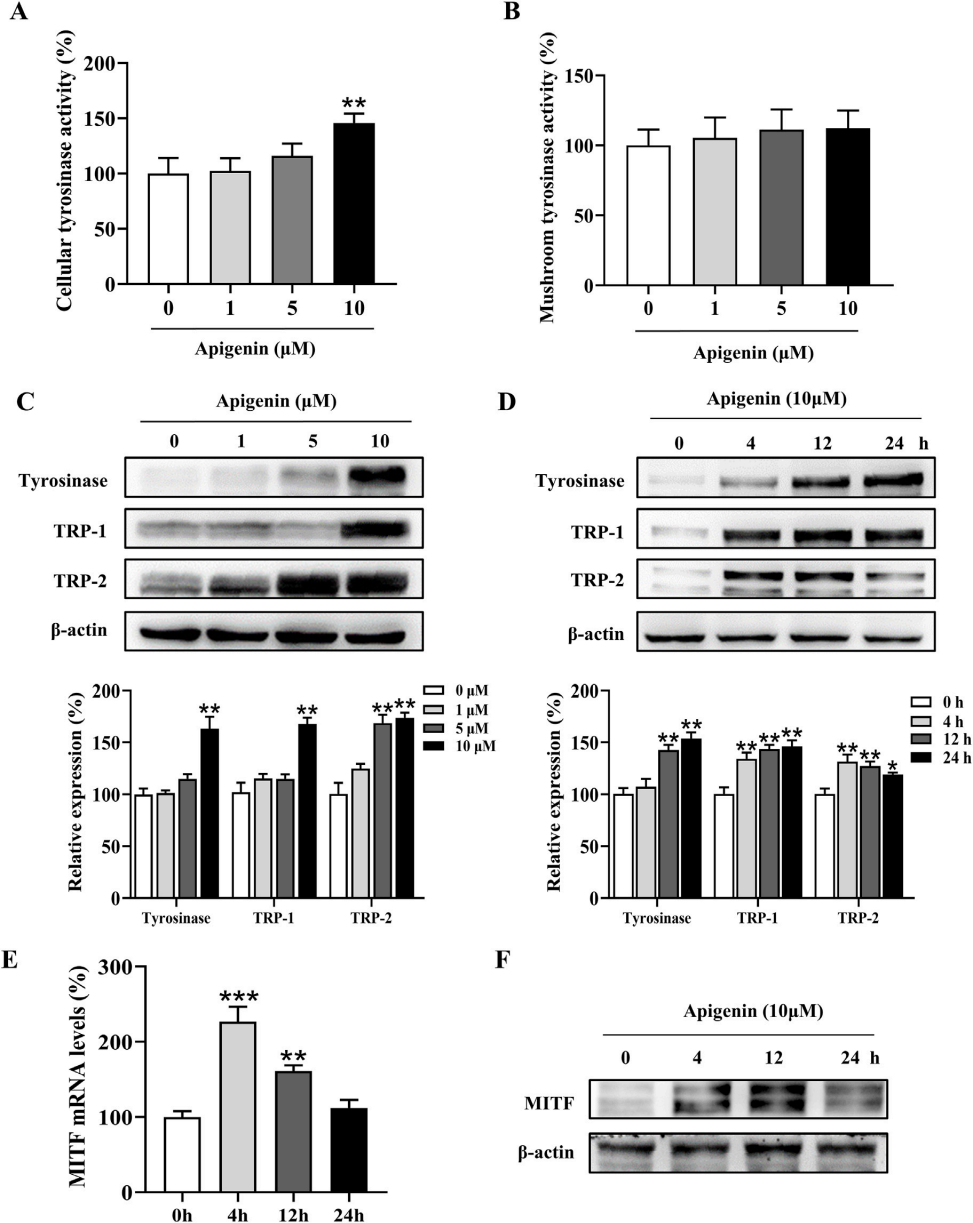
Apigenin increases cellular tyrosinase activity and expression of melanogenic proteins. **(A)** Cellular tyrosinase activity was measured after treatment with apigenin (0, 1, 5, or 10 μM). **(B)** Mushroom tyrosinase activity was measured in a cell-free assay after treatment with apigenin (0, 1, 5, or 10 μM). **(C)** HEMCs were treated with apigenin (0, 1, 5, or 10 μM) for 48 h, and Western blotting was performed to assess protein levels of melanogenic markers. **(D)** Time-dependent changes in the expression levels of various proteins after treatment with apigenin (10 μM) for the indicated periods. **(E)** HEMCs were treated with apigenin (10 μM) for 4 h, and RT-qPCR was performed to measure MITF gene expression. **(F)** HEMCs were treated with apigenin (10 μM) for different time points, followed by Western blotting to assess MITF protein levels. Data are expressed as means ± SEM (n = 3). *p < 0.05, **p < 0.01, and ***p < 0.001 vs. untreated cells. HEMCs, human epidermal melanocytes; MITF, Melanocytes inducing transcription factor.

Tyrosinase plays a central role in melanin production. To assess the effect of apigenin on intracellular tyrosinase activity, we performed an L-DOPA oxidation assay (Lv et al., 2020). As shown in Figure 2A, apigenin enhanced tyrosinase activity in HEMCs. We next evaluated the immediate effects of apigenin on tyrosinase activity using a mushroom tyrosinase-based enzymatic assay. Figure 2B shows that apigenin did not affect mushroom tyrosinase activity. To explore whether apigenin’s melanin-producing properties are linked to the expression levels of Tyrosinase, TRP-1, and TRP-2, we assessed their protein expression via Western blotting. As illustrated in Figures 2C,D, apigenin increased the expression of Tyrosinase, TRP-1, and TRP-2. Furthermore, we examined the expression of MITF, a key transcription factor involved in the survival, growth, and pigmentation of melanocytes. As shown in Figures 2E,F; Supplementary Figure S1, apigenin significantly upregulated MITF expression at both the transcriptional and protein levels in HEMCs. The mRNA level of Tyrosinase, TRP-1 and TRP-2 were significantly upregulated by apigenin (Supplementary Figure S2). These results suggest that apigenin enhances melanin production by upregulating the expression of Tyrosinase, TRP-1, TRP-2, and MITF.

### Apigenin promoted melanosome transport via Rab27a and Cdc42

**FIGURE 3:**
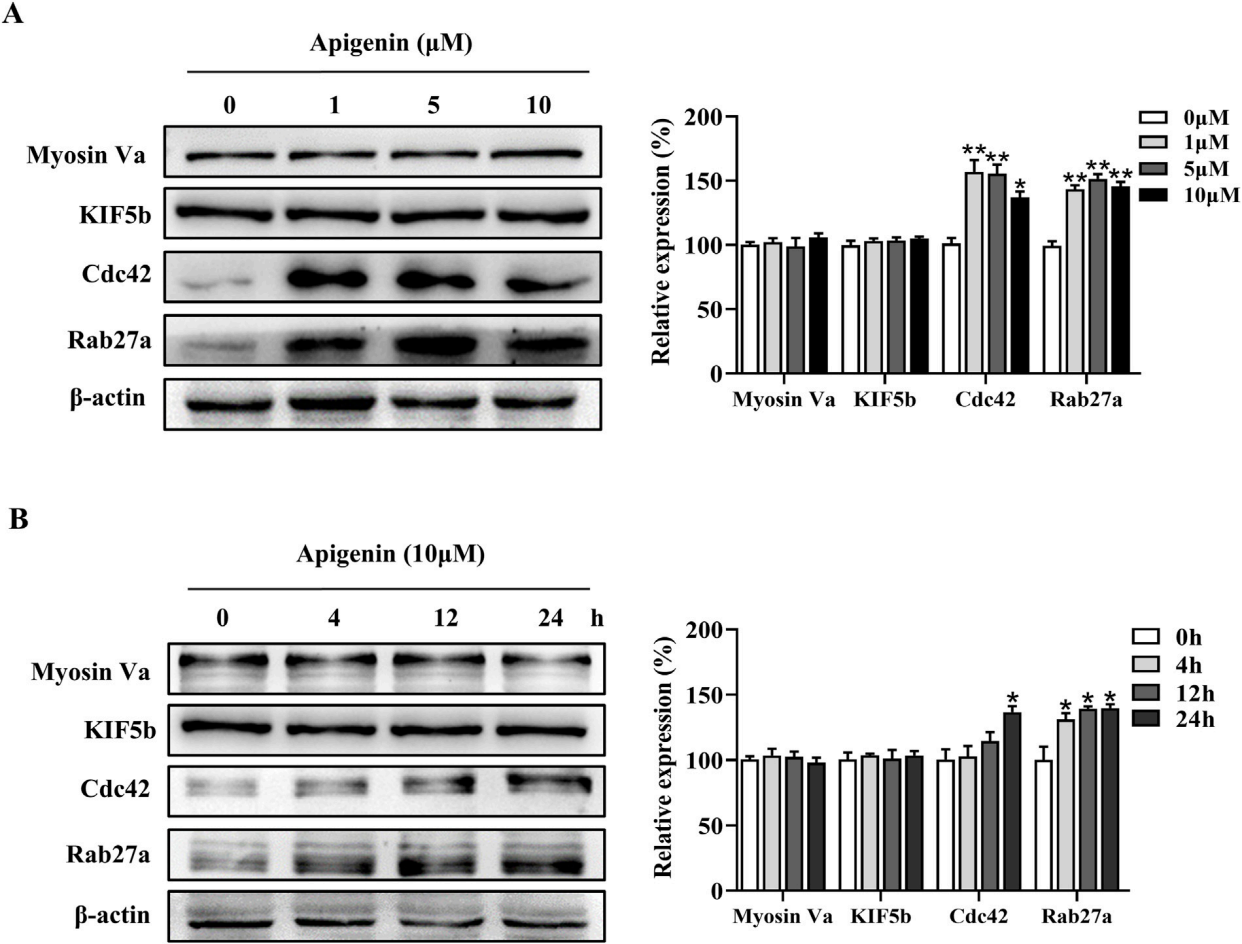
Apigenin promotes the expression of melanosome transport proteins in HEMCs. **(A)** HEMCs were treated with apigenin (0, 1, 5, or 10 μM) for 48 h, and Western blotting was performed to assess the expression levels of melanosome transport proteins. **(B)** Time-dependent changes in the expression levels of various melanosome transport proteins following treatment with apigenin (10 μM). Data are expressed as means ± SEM (n = 3). *p < 0.05, **p < 0.01 vs. untreated cells. HEMCs, human epidermal melanocytes.

Melanin synthesis occurs in the cell body of melanocytes and is subsequently transported to the dendrites before being transferred to adjacent keratinocytes for distribution (Ando et al., 2012). Apigenin increased the expression of Rab27a and Cdc42 in HEMCs (Figures 3A,B). In contrast, the expression levels of Kinesin superfamily proteins (KIF5b) and Myosin Va showed minimal changes compared to the control group (Figures 3A,B). The critical role of Rab27a and Cdc42 in apigenin-mediated melanocyte branching and melanosome transport was further confirmed using siRNAs against Rab27a and Cdc42. As shown in Supplementary Figure S3, Rab27a and Cdc42 knockdown reversed apigenin-induced melanogenic processes. These results suggest that apigenin enhances melanocyte branching and melanosome transport by upregulating the expression of the key proteins Rab27a and Cdc42.

### Apigenin increased pigmentation independent of the classic MC1R/cAMP/PKA signaling pathway

**FIGURE 4:**
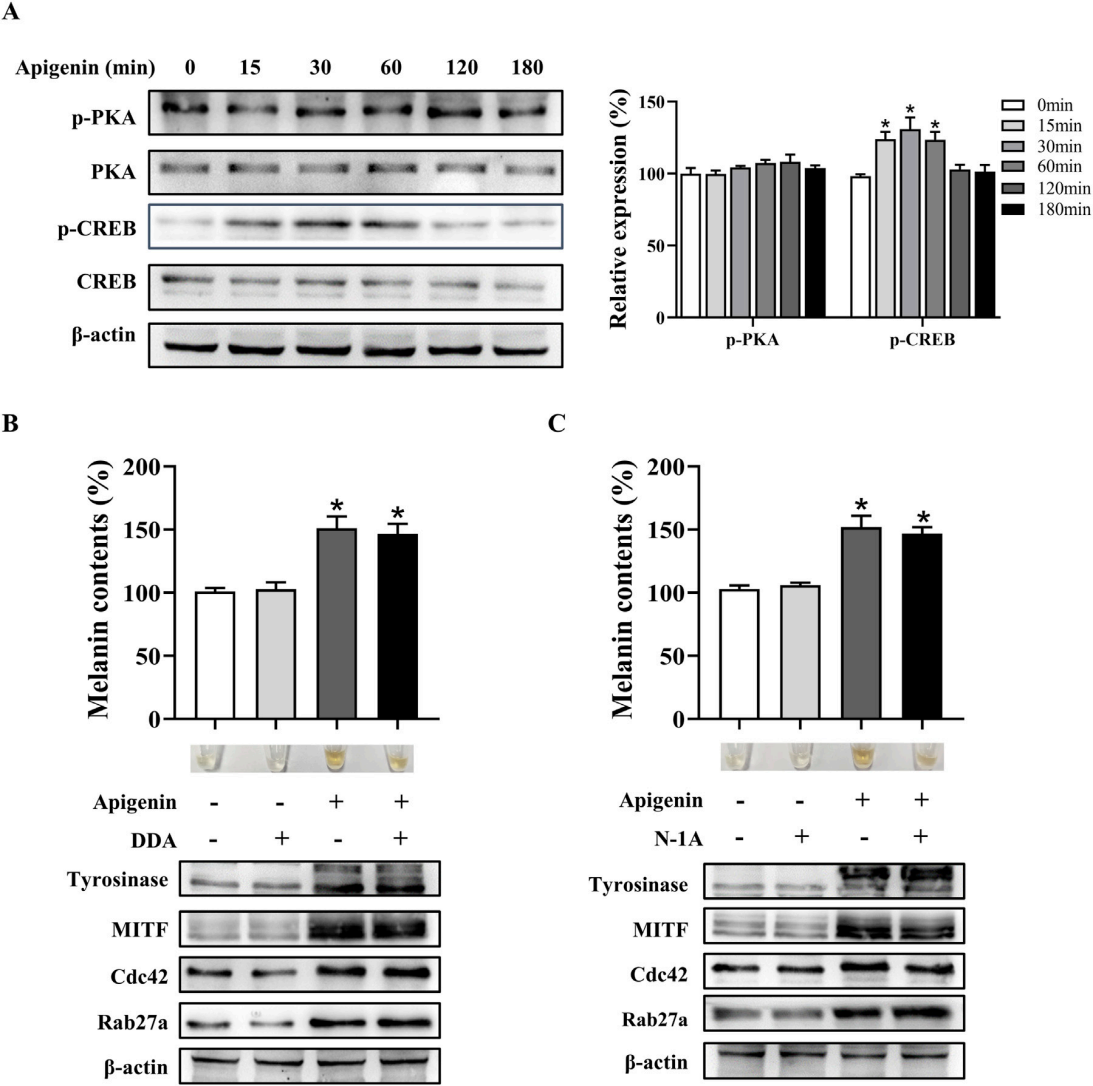
Apigenin promotes pigmentation independent of the classic MC1R/cAMP/PKA-pigmentation pathway in HEMCs. **(A)** HEMCs were treated with apigenin (10 μM) for the indicated time periods (0–180 min), and the phosphorylation of PKA and CREB was assessed by Western blotting. **(B)** HEMCs were pretreated with or without 10 μM DDA (an AC inhibitor) for 1 h before the addition of apigenin for 48 h. Melanin content, as well as the expression levels of Tyrosinase, MITF, Cdc42, and Rab27a, were measured as described previously. **(C)** HEMCs were pretreated with or without 10 μM N-1A (an MC1R inhibitor) for 1 h before apigenin treatment for 48 h. Melanin content and the expression levels of Tyrosinase, Cdc42, and Rab27a were measured as described previously. Data are expressed as the mean ± SEM (n = 3). *p < 0.05 vs. untreated cells. MC1R, melanocortin-1 receptor; cAMP, Cyclic Adenosine Monophosphate; PKA, protein kinase A; HEMCs, human epidermal melanocytes; CREB, cAMP response element-binding protein; AC, adenylate cyclase; MITF, Melanocytes inducing transcription factor; Cdc42, cell division cycle 42; Rab27a, Ras-related protein Rab-27a.

Melanogenesis is regulated by various signaling pathways, with the MC1R/cAMP/PKA pathway playing a critical role (D’mello et al., 2016). To investigate whether apigenin affects this pathway, we examined its impact on MC1R/cAMP/PKA signaling. Our results show that apigenin enhances CREB phosphorylation without significantly altering PKA phosphorylation (Figure 4A). Additionally, neither N-1A (a specific MC1R inhibitor) nor DDA (a specific AC inhibitor) suppressed the melanogenic effects of apigenin (Figures 4B,C). These findings suggest that apigenin-induced melanin synthesis is independent of the conventional MC1R/cAMP/PKA signaling pathway.

### Apigenin enhanced pigmentation via the c-KIT/Raf-1/MAPK signaling pathway

The c-KIT/Raf-1/MAPK pathway, parallel to the MC1R/AC/cAMP/PKA pathway, plays a pivotal role in melanocyte pigmentation and development through the activation of MITF. In the canonical c-KIT/Raf-1/MAPK signaling cascade, binding of SCF activates the c-KIT receptor, which in turn phosphorylates Raf-1 (Wood et al., 1992). This activation triggers MAPK, leading to a series of kinase reactions that phosphorylate and activate extracellular ERK, which subsequently activates RSK (Imokawa et al., 2000). Upon activation, MAPK translocates to the nucleus and phosphorylates CREB via RSK (Sato-Jin et al., 2008). Phosphorylated CREB (p-CREB), CREB-binding protein (CBP), and CREB-regulated co-activator 1 (CRTC1) work together in the nucleus to recruit transcriptional machinery and initiate MITF transcription, promoting melanogenesis. Additionally, activation of the c-KIT receptor leads to the phosphorylation of p38 mitogen-activated protein kinase (p38) and mitogen-and stress-activated protein kinase 1(MSK1), ultimately activating CREB (Niwano et al., 2018).

**FIGURE 5:**
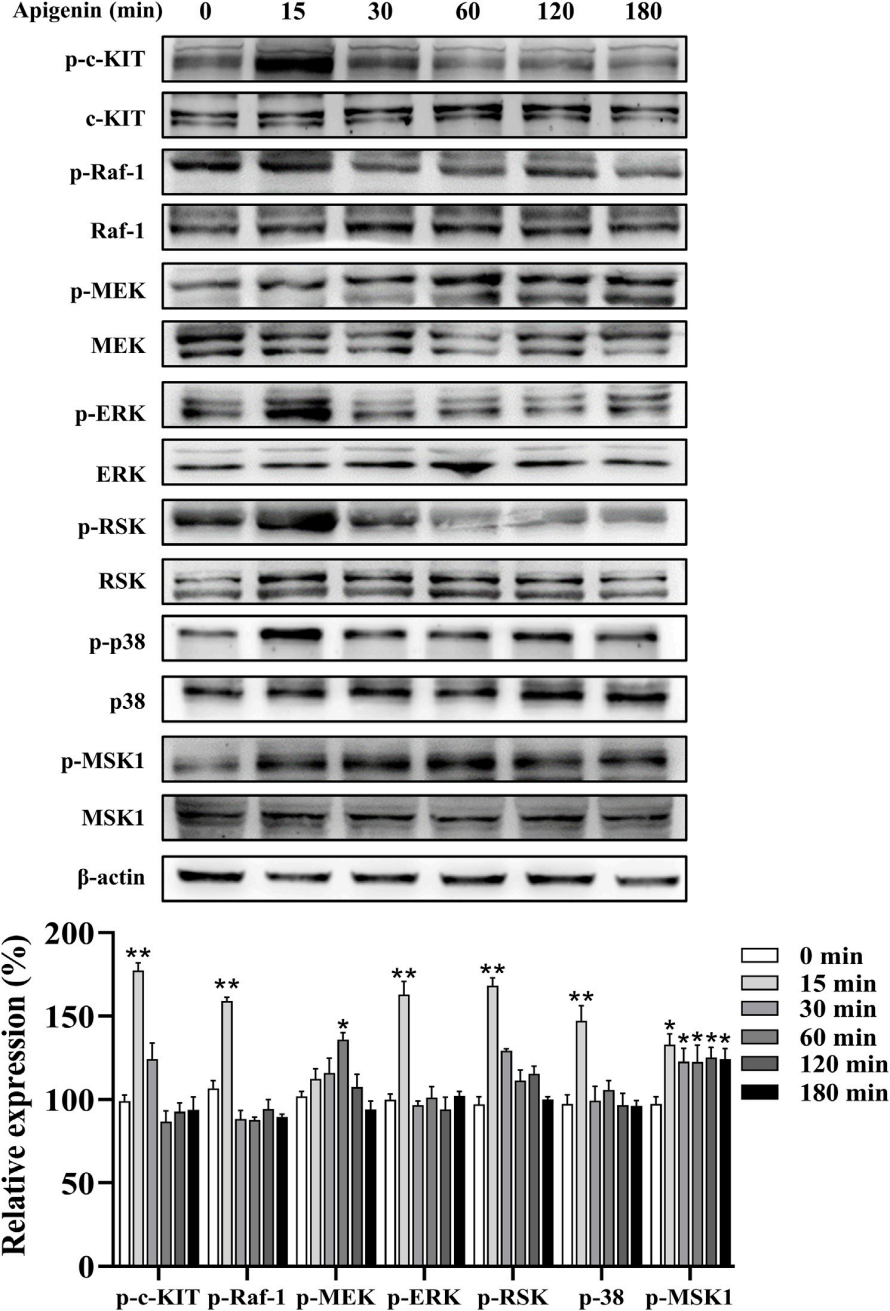
Apigenin activates the c-KIT/Raf-1/MAPK signaling pathway in HEMCs. HEMCs were treated with apigenin (10 μM) for the indicated time periods (0–180 min), and the phosphorylation levels of c-KIT, Raf-1, MEK, ERK, RSK, p38, and MSK1 were assessed by Western blotting. Data are expressed as means ± SEM (n = 3). *p < 0.05, **p < 0.01 vs. untreated cells. c-KIT, cellular-KIT; Raf-1, rapidly accelerated fibrosarcoma-1; MAPK, mitogen-activated protein kinase; HEMCs, human epidermal melanocytes; MEK, mitogen-activated protein kinase kinase; ERK, extracellular signal-regulated kinase; RSK, p90 ribosomal S6 kinase; p38, p38 mitogen-activated protein kinase; MSK1, mitogen-and stress-activated protein kinase 1.

**FIGURE 6:**
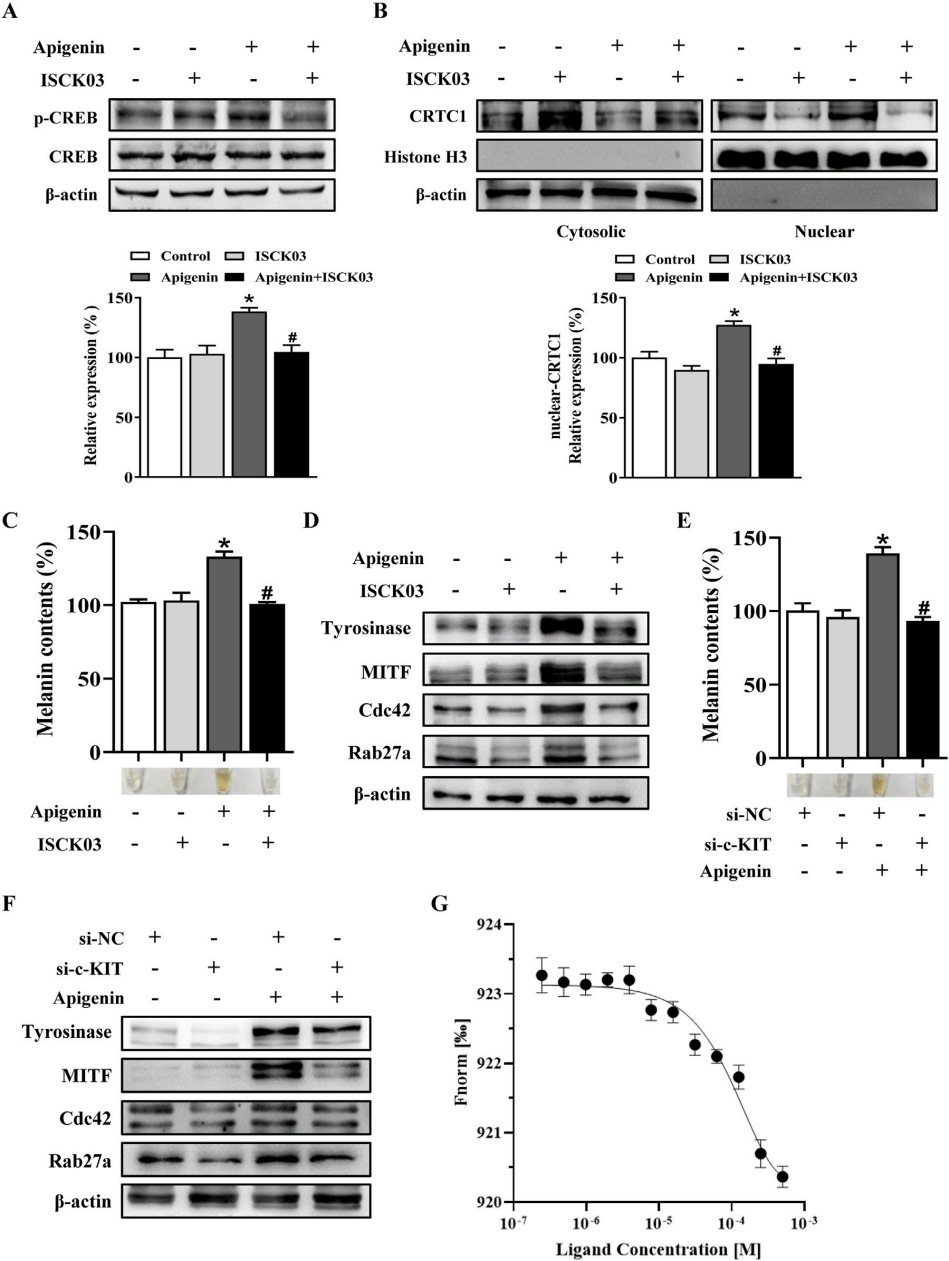
Apigenin promotes pigmentation through the c-KIT-CRTCs/CREB signaling pathway. **(A,B)** HEMCs were pretreated with or without 10 μM ISCK03 (c-KIT inhibitor) for 1 h before apigenin was added for an additional 1 h. Phosphorylated CREB (p-CREB) and nuclear levels of CRTC1 were measured by Western blotting. Histone H3 served as a reference for nuclear proteins, and β-actin served as a reference for cytosolic proteins. **(C,D)** HEMCs were pretreated with or without 10 μM ISCK03 for 1 h before apigenin was added for 48 h. Melanin content and the expression levels of Tyrosinase, MITF, Cdc42, and Rab27a were measured as described previously. **(E,F)** HEMCs were transfected with si-NC or si-c-KIT for 24 h, followed by apigenin (10 μM) treatment for 48 h. Melanin content and the expression levels of Tyrosinase, MITF, Cdc42, and Rab27a were measured as described previously. **(G)** Binding of c-KIT with apigenin was analyzed by microscale thermophoresis (MST). The binding curve represents data points from 3 measurements. The calculated Kd is 2.6 ± 0.14 µM. Data are expressed as means ± SEM (n = 3). *p < 0.05 vs. non-treated cells, ^#^p < 0.05 vs. apigenin-treated cells. c-KIT, cellular-KIT; CREB, cAMP response element-binding protein; CRTC, CREB-regulated co-activator; HEMCs, human epidermal melanocytes; MITF, Melanocytes inducing transcription factor; Cdc42, cell division cycle 42; Rab27a, Ras-related protein Rab-27a.

In this study, apigenin enhanced the phosphorylation of c-KIT, Raf-1, mitogen-activated protein kinase kinase (MEK), ERK, RSK, p38, and MSK1 in HEMCs (Figure 5). We next investigated whether the c-KIT/Raf-1/MAPK/RSK signaling cascade contributes to apigenin-induced melanogenesis. The c-KIT inhibitor ISCK03 effectively counteracted the stimulatory effects of apigenin on melanin production and the levels of p-CREB, p-ERK, p-RSK, Tyrosinase, MITF, Cdc42, and Rab27a (Figures 6A,C,D; Supplementary Figure S4A). Additionally, BI-D1870, an RSK inhibitor, reversed the stimulatory effects of apigenin on p-CREB, Tyrosinase, and MITF levels (Supplementary Figures S4B, S4C). Yun et al., reported that SCF activation promotes CRTC1 nuclear translocation (Yun et al., 2020), and our study confirmed that apigenin increased CRTC1 levels in the nucleus, while ISCK03 inhibited this translocation (Figure 6B). The critical role of c-KIT in apigenin-mediated melanogenesis was further confirmed using siRNAs against c-KIT. As shown in Figures 6E,F, Supplementary Figure S5, c-KIT knockdown reversed apigenin-induced melanogenesis and reduced Tyrosinase, MITF, Rab27a, and Cdc42 expressions in melanocytes. Microscale thermophoresis (MST) measurements were used to assess the direct interaction between apigenin and c-KIT. c-KIT exhibited a binding affinity to apigenin with a Kd of 2.6 µM at a target concentration of 50 nM (Figure 6G), confirming the moderate interaction between apigenin and c-KIT.

### Apigenin increased pigmentation in zebrafish

**FIGURE 7:**
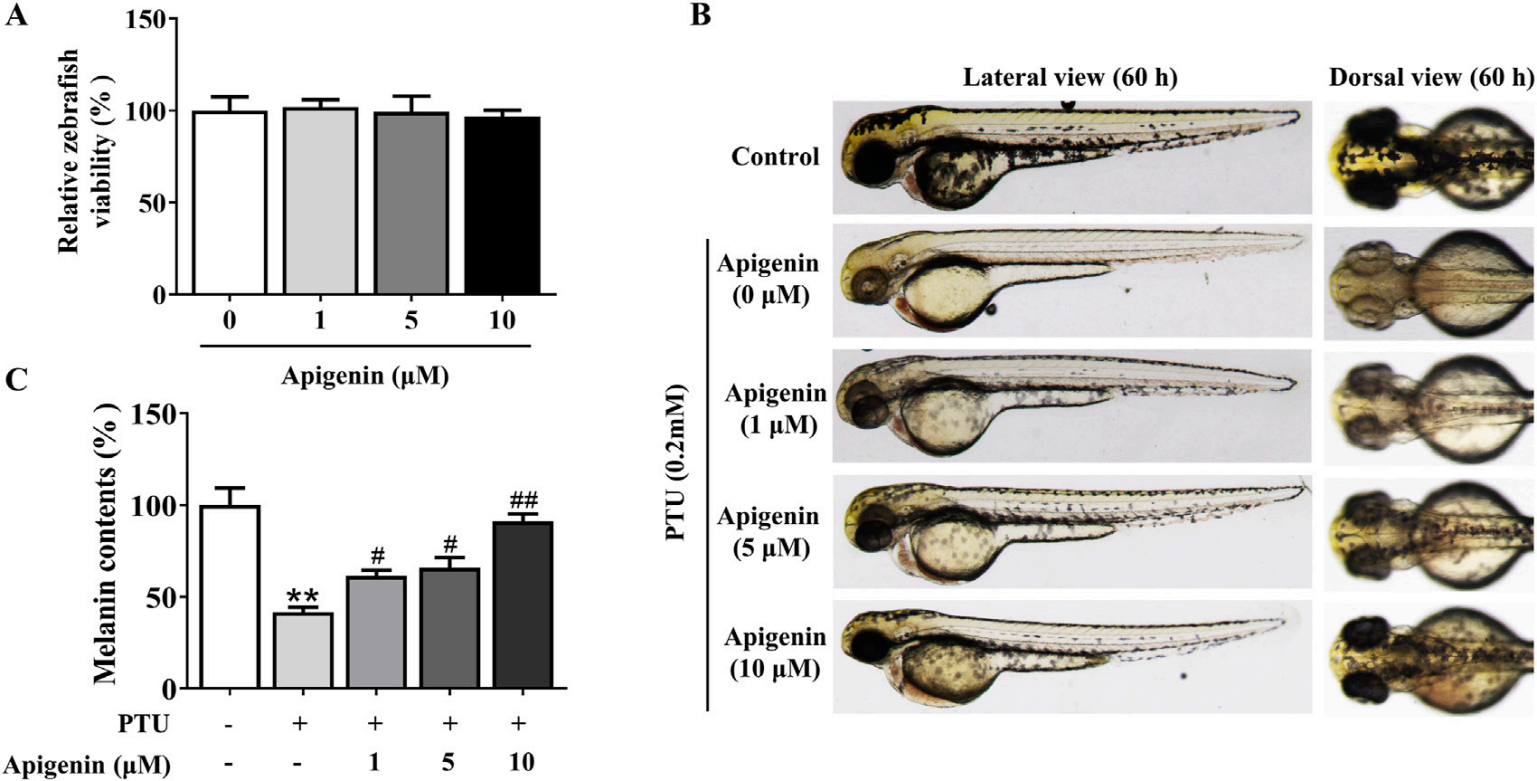
Apigenin increases pigmentation in zebrafish. **(A)** Zebrafish embryos were treated with various concentrations (1–10 μM) of apigenin for different time periods (35–60 h), and zebrafish viability was assessed. **(B)** Zebrafish embryos were treated with PTU (0.2 mM) for 6–35 h, followed by apigenin (0, 1, 5, or 10 μM) treatment in the embryo medium for 35– 60h. Zebrafish pigmentation was observed using a stereomicroscope. **(C)** Approximately 30 embryos were collected and dissolved in lysis buffer. After centrifugation, the melanin pigment was dissolved in 200 μL of NaOH working solution (1 mol/L, 10% DMSO) at 80°C for 2 h, and absorbance was measured at 405 nm. Data are expressed as means ± SEM (n = 3). **p < 0.01 vs. non-treated zebrafish, ^#^p < 0.05 and ^##^p < 0.01 vs. PTU-treated zebrafish. PTU, propylthiouracil; DMSO, Dimethyl Sulfoxide.

Using the zebrafish model, we further investigated the effect of apigenin on melanin production. Like humans and mice, zebrafish melanocytes originate from the neural crest, and the evolutionary conservation of the pathways governing melanocyte development and pigment production is evident across these species. To begin, we assessed the toxicity of apigenin in zebrafish. Based on the zebrafish survival rate, there was no significant embryotoxicity observed following apigenin treatment (below 10 μM) over 60 h (Figure 7A). PTU, a melanogenesis inhibitor commonly used in zebrafish, was employed to induce skin depigmentation and establish a model (Elsalini and Rohr, 2003). As shown in Figures 7B, C, apigenin significantly induced darkening in zebrafish previously depigmented by PTU exposure.

### Apigenin enhanced pigmentation and prevented UVB-induced DNA damage in human skin explants

**FIGURE 8:**
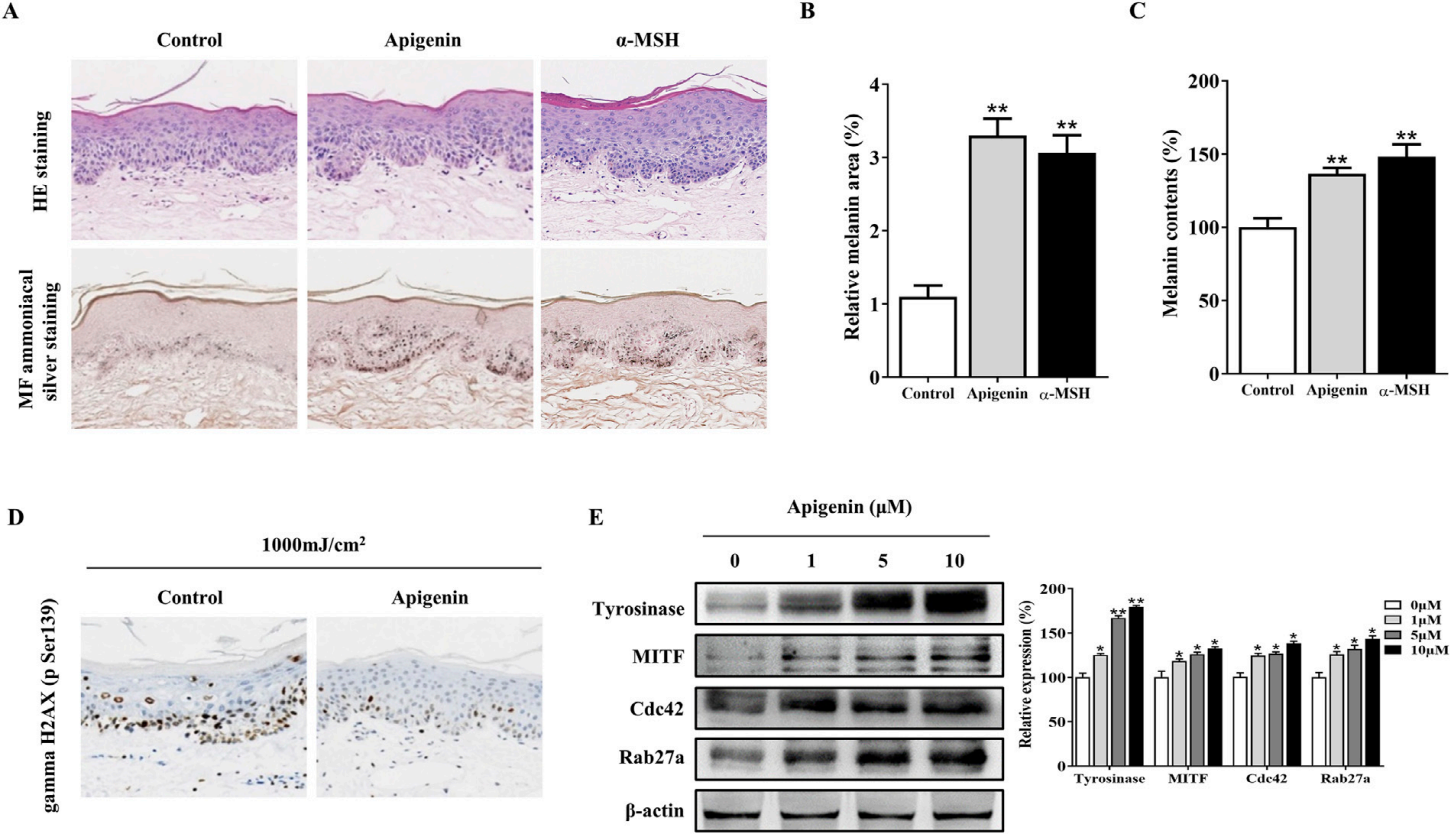
Apigenin induces pigmentation in human skin explants. **(A)** Human skin explants were treated with apigenin (10 μM) or α-MSH (100 nM) for 5 days. Top panel: H&E staining compared with the vehicle control (DMSO). Bottom panel: Masson-Fontana staining to detect melanin in human skin. **(B)** The ratio of melanin pigment to the cross-sectional area of human skin, as shown by Masson-Fontana staining. **(C)** Melanin content was measured in human skin explants treated with apigenin (10 μM) or α-MSH (100 nM) for 5 days. **(D)** Human skin explants were treated with apigenin for 5 days and exposed to UVB, followed by gamma H2AX (phospho S139) measurement using immunohistochemistry (IHC). **(E)** Western blotting was performed to assess related protein levels in human skin explants treated with apigenin for 5 days. Data are expressed as the mean ± SEM (n = 3). *p < 0.05, **p < 0.01 vs. non-treated groups. α-MSH, α-melanocyte-stimulating hormone; DMSO, Dimethyl Sulfoxide; UVB, Ultraviolet B.

Subsequently, we investigated the effects of apigenin on human skin explant pigmentation. The percentage of melanin pigment in the total cross-sectional area was determined by analyzing Masson-Fontana stained images of each section using ImageJ. As shown in Figures 8A, B, apigenin significantly increased the ratio of melanin pigment to the cross-sectional area of human skin explants, with no signs of cellular damage or inflammation observed in haematoxylin and eosin (H&E) staining. Notably, keratinocytes in treated skin exhibited supranuclear melanin caps, providing visual evidence of efficient melanin transfer into these cells. As shown in Figure 8C, apigenin significantly increased the overall melanin content in human skin explants. These findings suggest that apigenin not only enhances melanogenesis but also promotes the transfer of melanin to adjacent keratinocytes. Furthermore, we explored whether apigenin-induced skin pigmentation could offer protection against UVB-induced gamma H2AX induction, a marker for DNA double-strand breaks. Human skin samples were treated with apigenin and then exposed to UVB. As shown in Figure 8D, the pigmentation induced by apigenin exhibited protective effects against UVB-induced gamma H2AX induction. These findings strongly suggest that apigenin-induced pigmentation may serve as a protective barrier against UVB-induced DNA damage. Moreover, Western blotting and immunohistochemistry demonstrated that apigenin treatment elevated the expression of Tyrosinase, MITF, Cdc42, and Rab27a in human skin explants (Figure 8E; Supplementary Figure S6). To evaluate the role of key factors related to melanogenesis and melanosome transport in determining the color of human skin explants, the correlation between Tyrosinase, MITF, Cdc42, and Rab27a expressions and melanin content was examined. As shown in Supplementary Figure S7, apigenin-induced Tyrosinase, MITF, and Rab27a expression levels were correlated with melanin content in human samples. However, no correlation was observed between melanin content and Cdc42 protein expression in the present study.

## Discssion

Melanin plays a pivotal role in regulating cellular metabolism, suggesting that compounds capable of enhancing melanin production could potentially mitigate the detrimental effects of UV radiation on the skin, including damage and the risk of cancer (Allouche et al., 2021). Our study demonstrated that apigenin increased pigmentation in HEMCs, zebrafish, and human skin explants (Figures 1, 7, 8). Although apigenin did not directly inhibit tyrosinase activity, it significantly upregulated the expressions of Tyrosinase, TRP-1, and TRP-2, suggesting that apigenin promotes melanogenesis by increasing the levels of key enzymes involved in the process (Figure 2).

Skin pigmentation is regulated by complex mechanisms, including the movement of melanosomes within cells and their transfer to nearby keratinocytes. Masson-Fontana ammoniacal silver staining demonstrated that apigenin not only increased dendritic length but also enhanced dendritic branching and led to higher melanin accumulation in the pericellular area (Figure 1D). Melanosome trafficking involves both microtubule-based and actin-based motor systems. Specifically, KIF5b mediates long-range transport along microtubules, while Myosin Va facilitates final delivery near the cell periphery via actin filaments (Hara et al., 2000; Noguchi et al., 2014; Tian et al., 2021). Rab27a, a member of the Rab GTPase family, plays a pivotal role in actin-dependent melanosome tethering to the plasma membrane (Ohbayashi and Fukuda, 2012). Cdc42, a small Rho GTPase, is another key regulator known to orchestrate cytoskeletal remodeling and dendrite formation in melanocytes (Ohbayashi and Fukuda, 2012). Our results indicated that apigenin enhanced the expression of Cdc42 and Rab27a in HEMCs, while the expression levels of KIF5b and Myosin Va showed minimal variation compared with those in the control group (Figure 3). These findings suggest that apigenin promotes melanin movement along actin filaments and dendrite extension by modulating Cdc42 and Rab27a expression. However, further comprehensive research is needed to determine whether apigenin also affects melanosome movement along microtubules.

The regulation of melanogenesis involves intricate signaling cascades, with the MC1R/AC/PKA/CREB pathway playing a pivotal role (D’mello et al., 2016). Our study revealed that apigenin specifically induces CREB phosphorylation without affecting PKA phosphorylation (Figure 4A). Notably, the inhibition of MC1R and AC failed to reverse apigenin-induced melanin synthesis (Figures 4B,C). These findings suggest that apigenin-induced melanogenesis is independent of the conventional MC1R/cAMP/PKA signaling pathway. In addition to the MC1R/AC/PKA pathway, the c-KIT/Raf-1/MAPK pathway significantly contributes to melanocyte pigmentation and development by activating MITF. Our study demonstrates that apigenin significantly enhances the phosphorylation of c-KIT, Raf-1, MEK, ERK, RSK, p38, and MSK1 in HEMCs (Figure 5). Interestingly, both the c-KIT pathway inhibitor ISCK03 and c-KIT knockdown effectively reversed the stimulatory effect of apigenin on melanin production and the levels of p-CREB, nuclear-CRTC1, Tyrosinase, MITF, Cdc42, and Rab27a (Figure 6). The results of MST further confirmed the moderate interaction of apigenin with c-KIT (Figure 6G). Zhao et al., reported that apigenin inhibits the phosphorylation of ERK in human melanoma A375 and C8161 cell lines (Zhao et al., 2017). Lim et al., demonstrated that apigenin promotes the phosphorylation of ERK and RSK in human choriocarcinoma cells (Lim et al., 2016). These studies indicate that apigenin exerts a bidirectional regulatory effect on ERK activation in different cell types, which is consistent with our findings and supports the notion that apigenin promotes melanogenesis by activating the c-KIT/Raf-1/MAPK signaling pathway. In addition to activating Raf-1/ERK/RSK, apigenin also triggers the activation of the p38/MSK1 signaling pathway. Furthermore, Ye et al., reported that apigenin enhances melanogenesis via activation of the p38 MAPK pathway in B16 melanoma cells (Ye et al., 2011). However, our experiments with HEMCs yielded a different response. Specifically, two p38 inhibitors, PD169316 and SB203580, failed to inhibit apigenin-induced melanogenesis (data not shown). This discrepancy may be due to the distinct signaling pathways operating in melanoma cell lines compared to primary melanocytes. Consequently, further in-depth investigations are needed to comprehensively elucidate the underlying molecular mechanisms.

Finally, we investigated the effects of apigenin on pigmentation in living organisms. Zebrafish serve as an ideal model because their melanocytes originate from an embryonic lineage comparable to those in humans and mice, both deriving from the neural crest, and share regulatory pathways that control proliferation and pigmentation. The pigmentation patterns observed in zebrafish closely resemble those seen in human melanocytes. Our findings revealed that apigenin administration enhanced pigmentation in zebrafish (Figure 7). Skin pigmentation, particularly high levels of eumelanin, can protect against UVB-induced DNA damage in humans (D’orazio et al., 2006; Mujahid et al., 2017). This protective effect is attributed to the UV light-absorbing properties of eumelanin and its ability to mitigate oxidative stress radicals. Various strategies have been explored to enhance pigmentation, including the topical application of the cAMP agonist forskolin, which has shown efficacy in mice but faces challenges with skin penetration in humans (D’orazio et al., 2006). Afamelanotide, a synthetic version of α-MSH, is used to treat erythropoietic protoporphyria by promoting skin darkening, which protects against UV light sensitivity (Langendonk et al., 2015). Given that apigenin is a bioactive compound derived from dietary sources, modulation of pigmentation using apigenin represents a potential complementary approach (Figure 8). This strategy could be beneficial in addressing conditions characterized by hypopigmentation and in implementing preventive measures against skin cancer.

**FIGURE 9:**
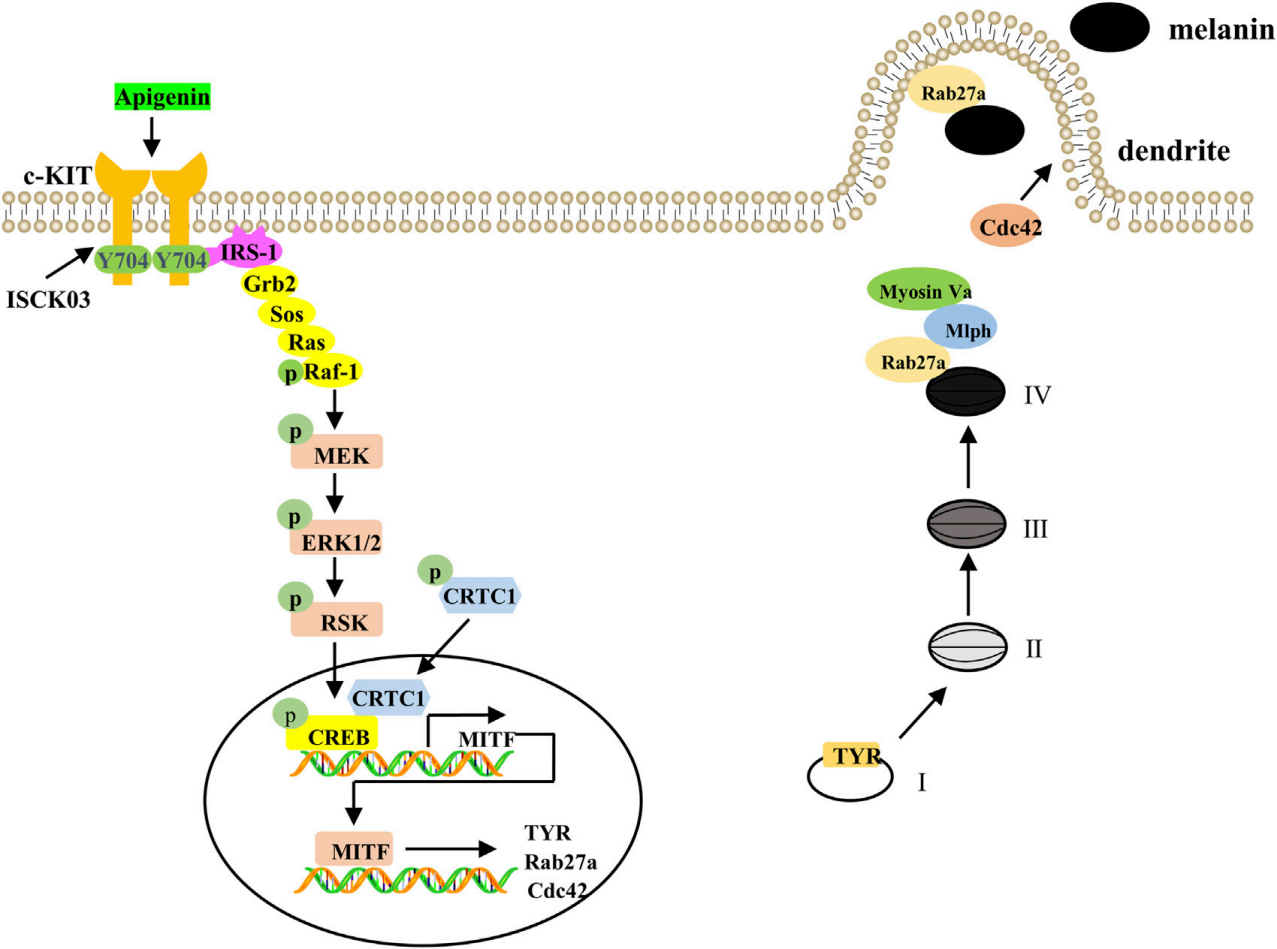
A proposed model showing that apigenin promotes pigmentation through the c-KIT/Raf-1/MAPK/CREB signaling pathway. Apigenin activates the c-KIT receptor, leading to phosphorylation of Raf-1. Once activated, Raf-1 phosphorylates and activates MAPK, resulting in CREB phosphorylation and subsequent nuclear translocation of CRTCs. Phosphorylated CREB, in association with CRTCs, promotes MITF transcription, which in turn induces the expression of Tyrosinase, Rab27a, and Cdc42. This cascade of events drives melanosome maturation and transport. Tyrosinase, TRP-1, and TRP-2 are key regulators of melanosome maturation. Cdc42 facilitates dendrite extension and filopodia formation, while Rab27a interacts with its effectors, Mlph and Myosin Va, to regulate actin-dependent melanosome transport and anchoring to the plasma membrane. c-KIT, cellular-KIT; Raf-1, rapidly accelerated fibrosarcoma-1; MAPK, mitogen-activated protein kinase; CRTC, CREB-regulated co-activator; CREB, cAMP response element-binding protein; MITF, Melanocytes inducing transcription factor; Cdc42, cell division cycle 42; Rab27a, Ras-related protein Rab-27a; TRP-1, tyrosinase-related protein-1; TRP-2, tyrosinase-related protein-2.

Overall, our results demonstrate that apigenin promotes melanogenesis, melanocyte dendricity, and melanosome transport through the c-KIT/Raf-1/ERK/RSK/CREB/MITF signaling pathway (Figure 9). These findings collectively highlight the potential of apigenin to enhance pigmentation in human melanocytes, zebrafish, and skin explants. Notably, our study reveals that apigenin-induced pigmentation serves as a protective shield against UVB-induced DNA damage, suggesting that apigenin is a promising and safe agent for treating hypopigmentation-related skin disorders. However, further *in vivo* validation and safety evaluation are needed, and future studies should explore additional signaling pathways and assess the clinical applicability of apigenin-based therapies.

## Data Availability

The original contributions presented in the study are included in the article/Supplementary Material, further inquiries can be directed to the corresponding author.
