# Supplementary material for: Apigenin promotes melanogenesis and melanosome transport through the c-KIT/Raf-1/MAPK/CREB pathway in HEMCs
Source: Front Pharmacol. 2025 Apr 28;16:1572878. doi: 10.3389/fphar.2025.1572878 (PMC12066314; doi:10.3389/fphar.2025.1572878)
Supplement: Supplementary file 1 [file Presentation1.pptx]

## Slide 1
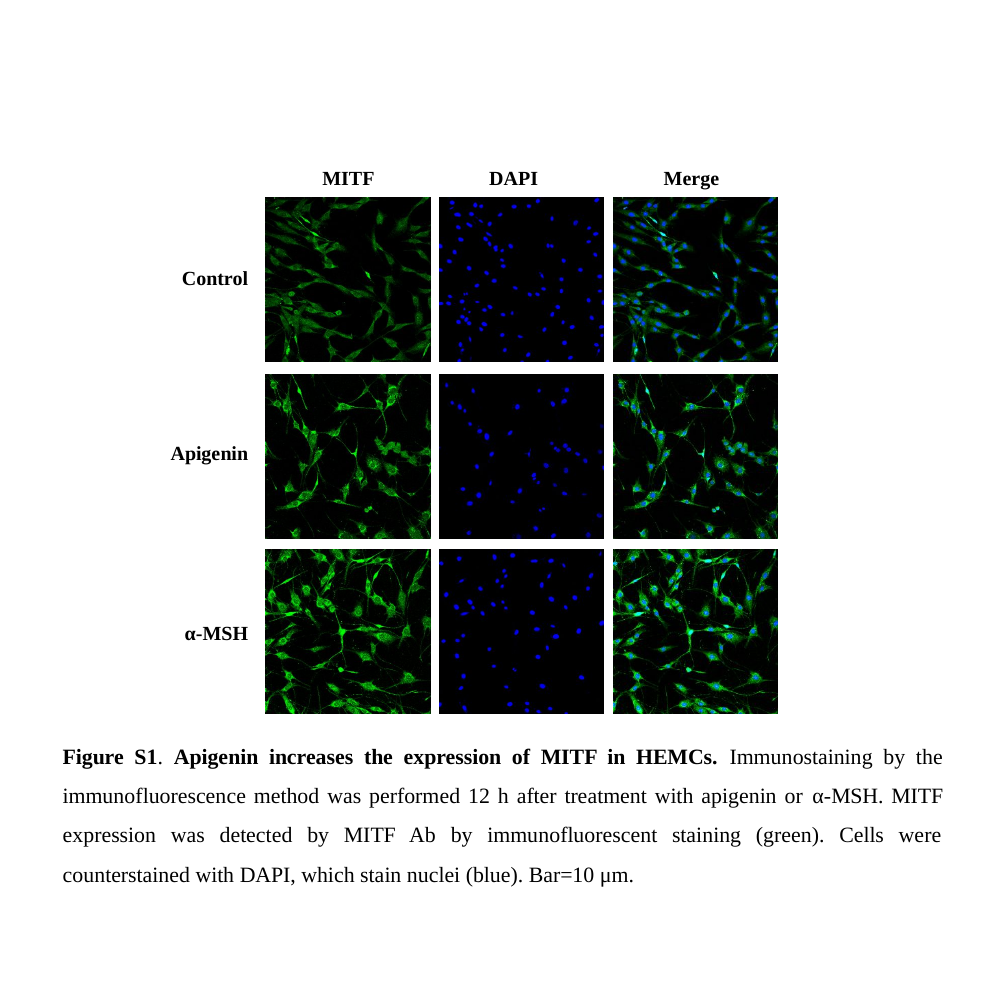

MITF
DAPI
Merge
Control
Apigenin
α-MSH
Figure S1. Apigenin increases the expression of MITF in HEMCs. Immunostaining by the immunofluorescence method was performed 12 h after treatment with apigenin or α-MSH. MITF expression was detected by MITF Ab by immunofluorescent staining (green). Cells were counterstained with DAPI, which stain nuclei (blue). Bar=10 μm.

## Slide 2
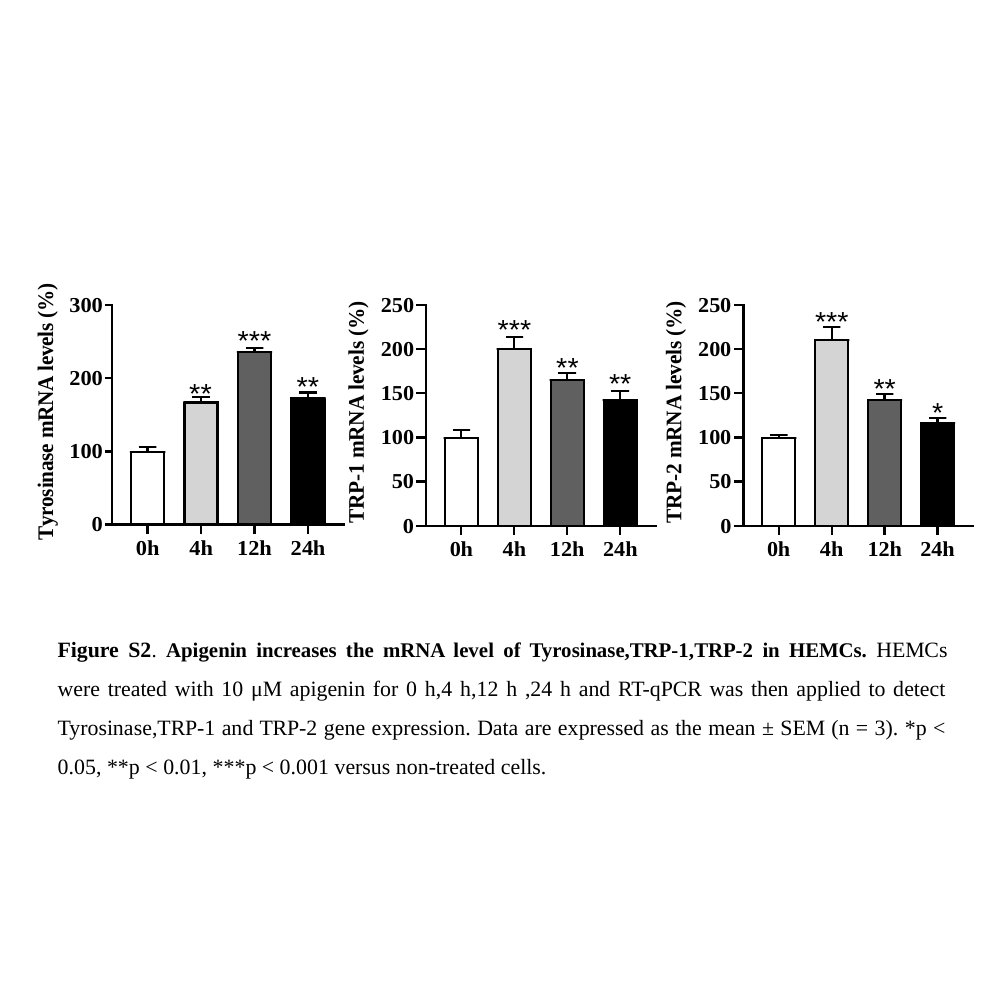

Figure S2. Apigenin increases the mRNA level of Tyrosinase,TRP-1,TRP-2 in HEMCs. HEMCs were treated with 10 μM apigenin for 0 h,4 h,12 h ,24 h and RT-qPCR was then applied to detect Tyrosinase,TRP-1 and TRP-2 gene expression. Data are expressed as the mean ± SEM (n = 3). *p < 0.05, **p < 0.01, ***p < 0.001 versus non-treated cells.

## Slide 3
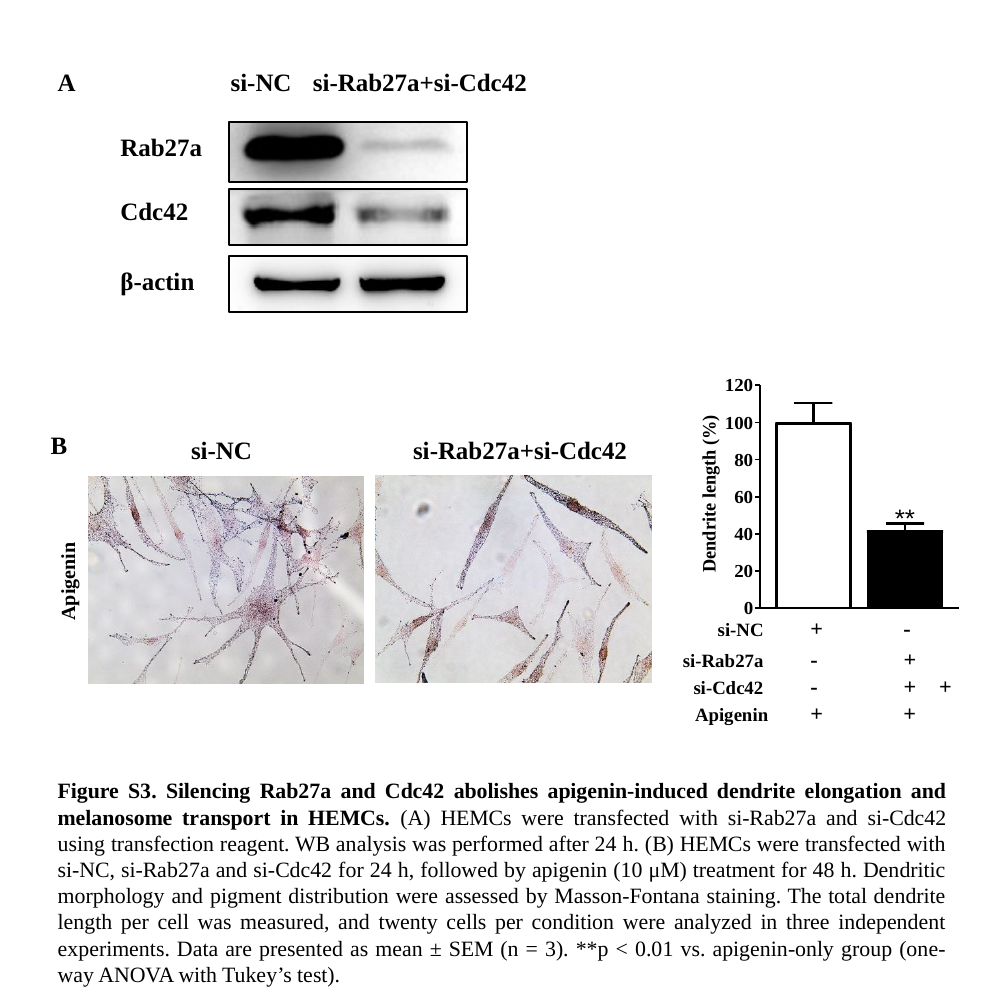

A
si-NC
si-Rab27a+si-Cdc42
Rab27a
Cdc42
β-actin
B
si-NC
si-Rab27a+si-Cdc42
Apigenin
+ -
si-NC
- +
si-Rab27a
- + +
si-Cdc42
+ +
Apigenin
Figure S3. Silencing Rab27a and Cdc42 abolishes apigenin-induced dendrite elongation and melanosome transport in HEMCs. (A) HEMCs were transfected with si-Rab27a and si-Cdc42 using transfection reagent. WB analysis was performed after 24 h. (B) HEMCs were transfected with si-NC, si-Rab27a and si-Cdc42 for 24 h, followed by apigenin (10 μM) treatment for 48 h. Dendritic morphology and pigment distribution were assessed by Masson-Fontana staining. The total dendrite length per cell was measured, and twenty cells per condition were analyzed in three independent experiments. Data are presented as mean ± SEM (n = 3). **p < 0.01 vs. apigenin-only group (one-way ANOVA with Tukey’s test).

## Slide 4
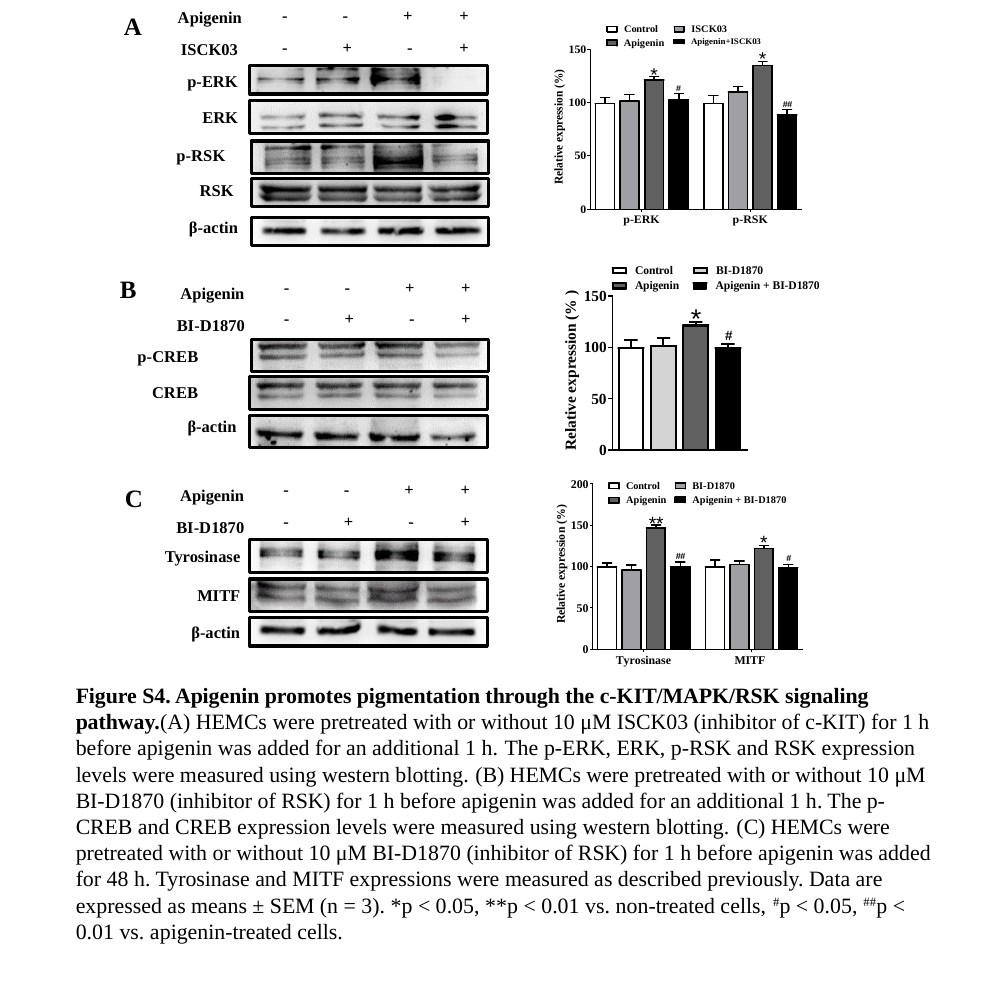

Apigenin
- - + +
A
ISCK03
- + - +
p-ERK
ERK
p-RSK
RSK
β-actin
B
- - + +
Apigenin
- + - +
BI-D1870
p-CREB
CREB
β-actin
- - + +
C
Apigenin
- + - +
BI-D1870
Tyrosinase
MITF
β-actin
Figure S4. Apigenin promotes pigmentation through the c-KIT/MAPK/RSK signaling pathway.(A) HEMCs were pretreated with or without 10 μM ISCK03 (inhibitor of c-KIT) for 1 h before apigenin was added for an additional 1 h. The p-ERK, ERK, p-RSK and RSK expression levels were measured using western blotting. (B) HEMCs were pretreated with or without 10 μM BI-D1870 (inhibitor of RSK) for 1 h before apigenin was added for an additional 1 h. The p-CREB and CREB expression levels were measured using western blotting. (C) HEMCs were pretreated with or without 10 μM BI-D1870 (inhibitor of RSK) for 1 h before apigenin was added for 48 h. Tyrosinase and MITF expressions were measured as described previously. Data are expressed as means ± SEM (n = 3). *p < 0.05, **p < 0.01 vs. non-treated cells, #p < 0.05, ##p < 0.01 vs. apigenin-treated cells.

## Slide 5
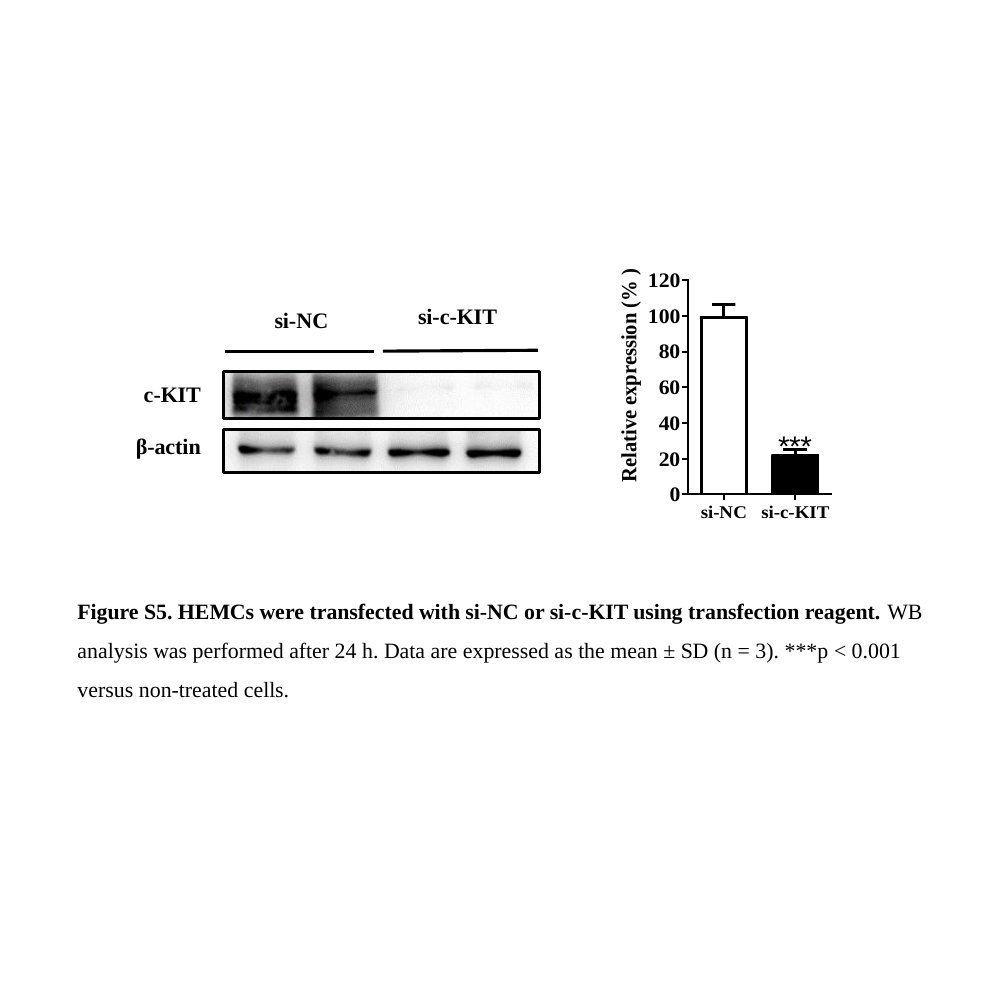

si-c-KIT
si-NC
c-KIT
β-actin
Figure S5. HEMCs were transfected with si-NC or si-c-KIT using transfection reagent. WB analysis was performed after 24 h. Data are expressed as the mean ± SD (n = 3). ***p < 0.001 versus non-treated cells.

## Slide 6
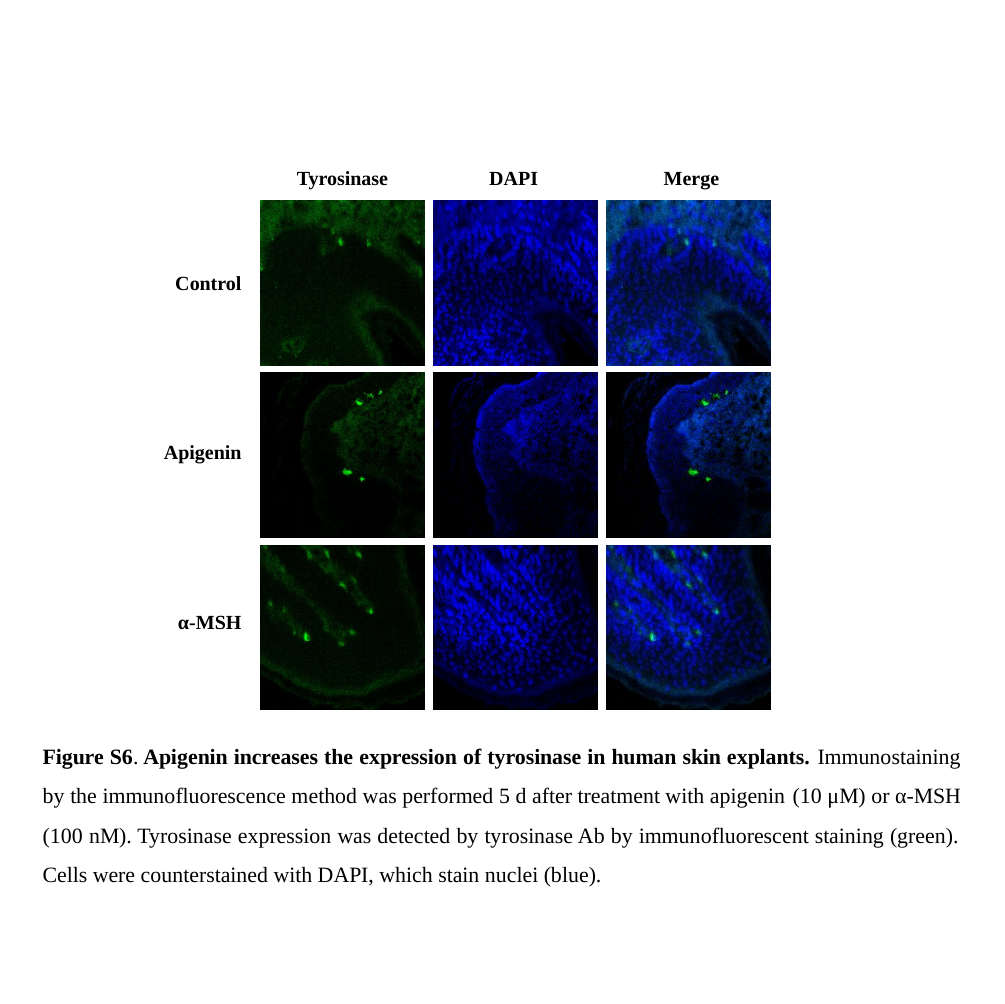

Tyrosinase
DAPI
Merge
Control
Apigenin
α-MSH
Figure S6. Apigenin increases the expression of tyrosinase in human skin explants. Immunostaining by the immunofluorescence method was performed 5 d after treatment with apigenin (10 μM) or α-MSH (100 nM). Tyrosinase expression was detected by tyrosinase Ab by immunofluorescent staining (green). Cells were counterstained with DAPI, which stain nuclei (blue).

## Slide 7
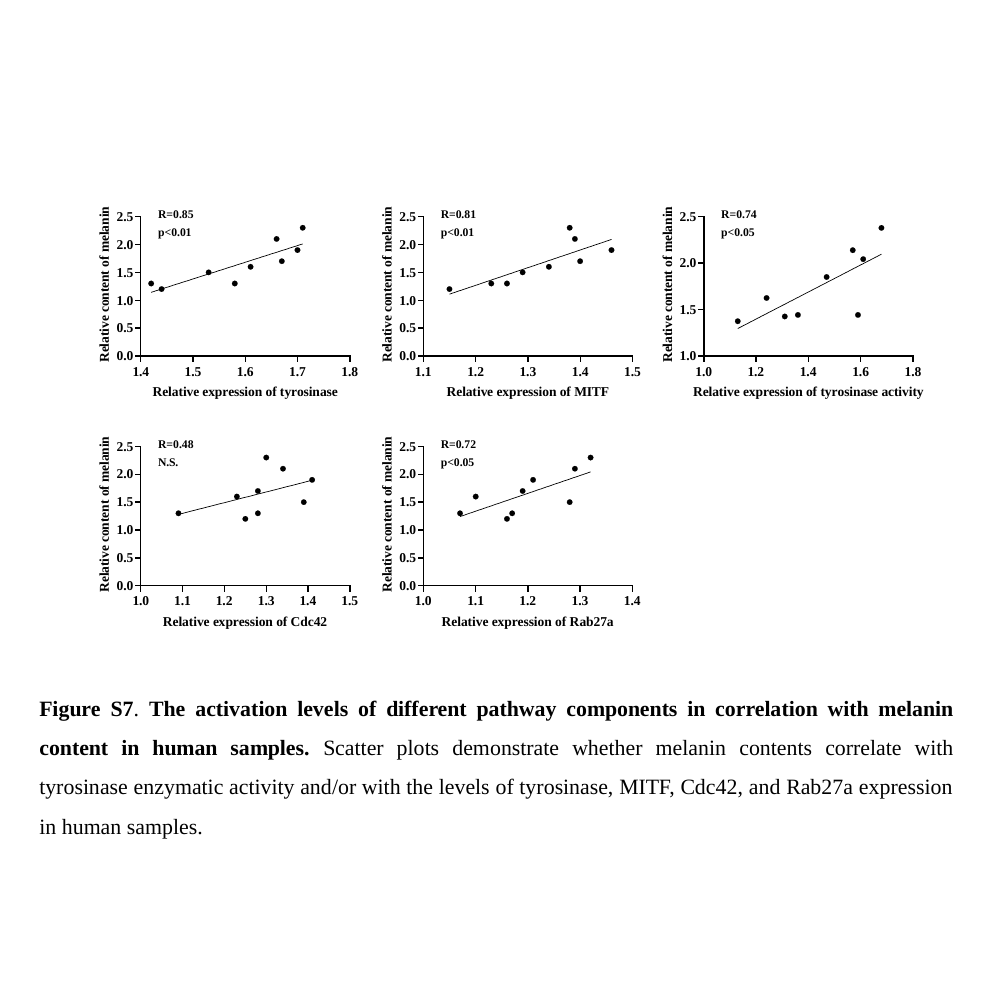

Figure S7. The activation levels of different pathway components in correlation with melanin content in human samples. Scatter plots demonstrate whether melanin contents correlate with tyrosinase enzymatic activity and/or with the levels of tyrosinase, MITF, Cdc42, and Rab27a expression in human samples.
