# Supplementary material for: Apigenin promotes melanogenesis and melanosome transport through the c-KIT/Raf-1/MAPK/CREB pathway in HEMCs
Source: Front Pharmacol. 2025 Apr 28;16:1572878. doi: 10.3389/fphar.2025.1572878 (PMC12066314; doi:10.3389/fphar.2025.1572878)
Supplement: Supplementary file 2 [file DataSheet1.docx]

1. The full scans of the entire original gels displayed in our manuscript

<https://www.jianguoyun.com/p/DRSbGx0Qy6SKCxjRjfYFIAA>

1. The original files for any microscopy images

<https://www.jianguoyun.com/p/DZeHXsEQy6SKCxjQjfYFIAA>

1. Raw data for the information presented in our figures

https://www.jianguoyun.com/p/Dfri8AwQy6SKCxjTjfYFIAA
